# Freespace “twistronics”: moiré structured light for optical supertopologies

**DOI:** 10.1038/s41377-026-02533-w

**Published:** 2026-11-06

**Authors:** Vasu Dev, Yijie Shen

**Affiliations:** 1 Centre for Disruptive Photonic Technologies, School of Physical and Mathematical Sciences, Nanyang Technological University, Singapore, 637371 Singapore; 2 Present Address: Department of Physics, School of Advanced Sciences (SAS), Vellore Institute of Technology (VIT), Vellore, Tamil Nadu 632014 India; 3 School of Electrical and Electronic Engineering, Nanyang Technological University, Singapore, 639798 Singapore

**Keywords:** Optical manipulation and tweezers, Nanophotonics and plasmonics

## Abstract

Moiré superlattices of twisted bilayer 2D materials creating nontrivial physical effects have recently revolutionized diverse subjects across materials, optoelectronics, nanophotonics, and beyond. Here, we present moiré superlattices in higher-dimensional freespace optics, where the basic twisted lattice is not a layer of 2D material, but a propagating structured light field with periodic topological textures. The moiré structured light with flexibly tuned twist angle can generate and transit among a rich set of topologies, including skyrmionium bags, skyrmion bag superlattices, skyrmion clusters, and quasicrystals, with controlled symmetry, most of which were not discovered in any other systems. Importantly, our freespace moiré textures maintain their topologies over long-distance propagation with nondiffractive behavior and topological robustness against perturbations and obstacles. Our work unlocks higher dimensions of moiré photonics to manipulate super-resilient topologies and address the challenges of robust information transfer and encryption.

## Abstract (web summary)

We extend “twistronics” from 2D materials to freespace optics. By twisting moiré structured light, we create optical topological superlattices with super-robust, long-distance, nondiffractive propagation, and multi-dimensions to reconfigure skyrmionic topologies.

## Graphical abstract

**Figure:**
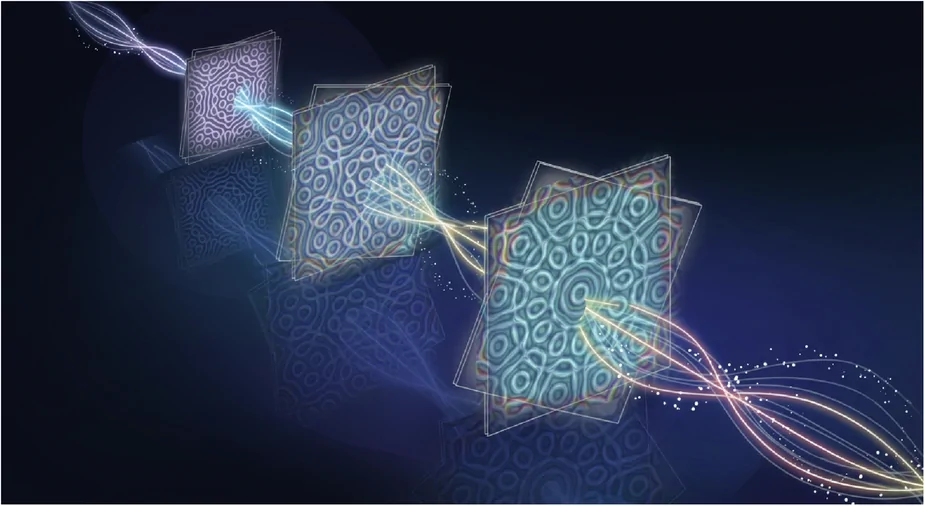

## Introduction

The interference of two periodic lattices with a twist induces a moiré superlattice pattern, which has transitioned from everyday textiles to the scientific frontier, called “twistronics”^1^. Remarkably, the moiré patterns on the twisted bilayer of atomically thin 2D materials can generate entirely novel physical properties with “1 + 1 ≫ 2” scheme, e.g., the superconductivity^2,3^, correlated insulation^4^, tunable spin-polarized phases^5^, flat-band and quantum geometry^6^ in twisted bilayer and multilayer graphene. Moreover, twistronics has been applied to a variety of advanced materials, where specific twist angles known as “magic” angles can radically alter the electronic and photonic properties of materials^7^. For instance, the excitonic devices with twisted van der Waals heterostructures^8^, photonic hyperbolic polaritons excited on twisted *α*-MoO_3_^9^, optical Hall effect enabled by spiral multilayer WS_2_^10^. Twisted photonic crystals can be used to localize light fields^11^ and enhance reconfigurable and singular nanolasers^12,13^. Recently, twistronics was also extended to hydrodynamics to control energy localization in fluid^14^, and also to surface plasmon polariton to create nontrivial topological textures of evanescent waves on metal surface^15^.

However, all these twisted moiré patterns rely on a static material as basis, and the related phenomena occur primarily at near field and low-dimensional surfaces. The moiré phenomena in high-dimensional, dynamic, and reconfigurable physical systems are unexplored. On the other hand, the investigation of novel high-dimensional topological structures, including and beyond skyrmions, has become a central pursuit across diverse physical systems aiming at achieving robust and reconfigurable information carriers, including antiferromagnetic merons^16^, magnetic skyrmion bundles^17^, hopfions^18^, torons in liquid crystals^19^, optical spin torons^20^, optical skyrmion and meron lattices^21–26^, optical hopfion crystals^27^, and other complex topologies observed in both light and matter systems^28–31^.

Here, we extend the concept of “twistronics” into freespace propagating structured light lattices, as a flexible platform to manipulate a broad family of nontrivial topological structures of light, which we called “optical supertopologies”. Important features of the supertopologies include: (1) Supertopologies are generated by superimposing two volumes of topological optical lattices with flexibly tuned twist angle, so as to create novel topologies, e.g. skyrmionium bags, skyrmionium-bag superlattices, skyrmion clusters, skyrmion-cluster superlattices, also including recently discovered topologies such as topological quasicrystals^32–35^ and skyrmion bags^15^ in plasmonic systems. At certain commensurate twist angles, the resulting moiré field forms periodic arrangement of skyrmionium bags (or skyrmion clusters), which we refer to as skyrmionium-bag (or skyrmion-cluster) superlattices. Importantly, the digital programmability of constituent lattices and their relative twist angle allow flexible switching among a broad range of supertopologies without the need for fabricating a new physical sample, in contrast to previous electronic or plasmonic twistronic platforms. (2) The supertopologies can propagate in free space over extended distances without diffraction, maintaining both their geometric scale and topological structure. (3) Supertopologies are intrinsically resilient to strong perturbations upon propagation and exhibit self-healing of their topological structures even damaged with obstacles.

## Results

### Twisted topological lattices

We first develop a method to generate topological optical lattices with high phase stability and accuracy and then apply inter-lattice twist to obtain nontrivial high-dimensional topologies. The lattices are generated through the principle of *N*-wave interference, in which two structured light lattices with binary and helical phase distributions and orthogonal polarizations are superimposed. In the Fourier domain, such lattices can be expressed as an array of *N* discrete point sources, which is experimentally challenging to realize. Therefore, to simplify, we approximate intensity of each point source with a Gaussian distribution. Therefore, the topological lattice in the Fourier domain can be expressed as:

1 $${\widetilde{U}}\,=\,\mathop{\sum }\limits_{j=1}^{N}{e}^{-\frac{{\left(x-{\alpha }_{j}\right)}^{2}+{\left(y-{\beta }_{j}\right)}^{2}}{{\omega }_{0}^{2}}}\left[\hat{{{\boldsymbol{e}}}_{{\boldsymbol{r}}}}+\mu {e}^{i\left({\theta }_{0}+{\phi }_{j}\right)}\hat{{{\boldsymbol{e}}}_{{\boldsymbol{l}}}}\right]$$

Where $\mu ={A}_{2}/{A}_{1}$ and ${A}_{1}$ and ${A}_{2}$ denote the amplitudes of right- and left-circularly polarized (RCP and LCP) light fields, $\hat{{{\boldsymbol{e}}}_{{\boldsymbol{r}}}}$ and $\hat{{{\boldsymbol{e}}}_{{\boldsymbol{l}}}}$, respectively. The coordinates of the *j-*th Gaussian beamlet are given by $\left({\alpha }_{j},{\beta }_{j}\right)=\gamma \left(\cos {\delta }_{j},\sin {\delta }_{j}\right)$, where $\gamma =d/\sqrt{2(1-\cos \left(\frac{2\pi }{N}\right))}$ and ${\delta }_{j}=\pi (2j-1)/N$ with $j=1,\,2,\,\ldots \ldots ,N$. The parameter d represents the center-to-center separation between neighboring Gaussian beamlets with the same beam waist ${\omega }_{0}$. The discrete helical phase of each beamlet in LCP field is defined as ${\phi }_{j}=m\left[\frac{2\pi \left(j-1\right)}{N}\right]$, where *m* is the topological charge, and *θ*_0_ is the global phase difference between the RCP and LCP. Following this formulation, we generate topological optical lattices exhibiting three characteristic symmetries: hexagonal C_6_ skyrmionium lattices (*N* = 6), square C_4_ skyrmion lattices (*N* = 4), and triangular C_3_ meron lattices (*N* = 3) (see the Extended Data Fig. E1).

**Fig. 1:**
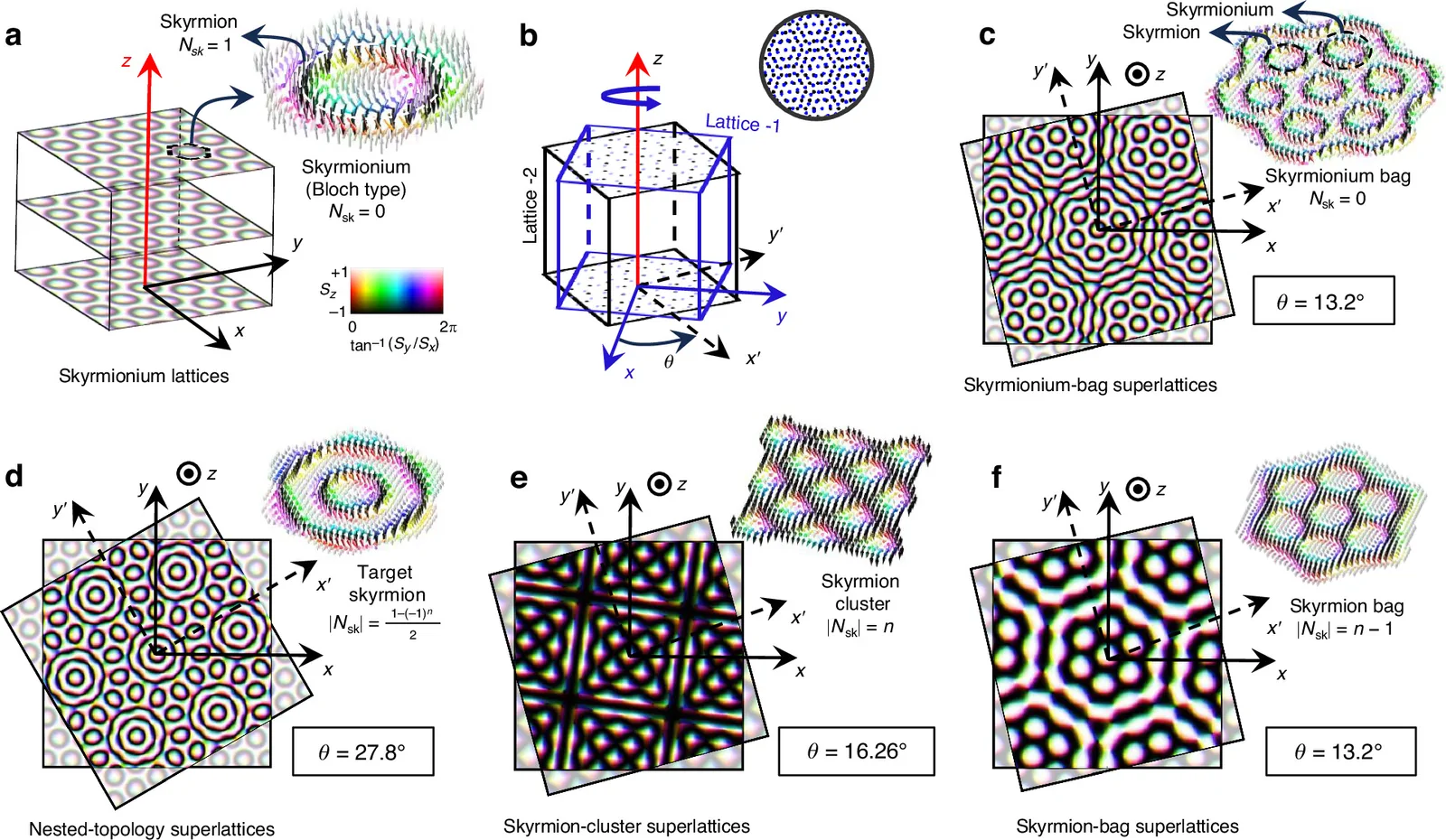
Concept of freespace twistronics and supertopologies. **a** A basic freespace topological optical lattice field, which hosts C_6_ symmetric skyrmionium lattices at each *x*-*y* plane and propagates along *z*-axis with nondiffraction. The inset highlights the skyrmionium texture as a unit cell at a transverse plane, where the inner skyrmion with unit topology is marked by dashed line. **b** Schematic of the moiré pattern formed by superimposing two topological optical lattice fields (blue and black, where the dots represent centers of unit cells) with a twisted angle *θ* about the common *z*-axis. The inset shows a schematic moiré superlattice pattern at a transverse plane. **c** The resultant moiré structured light formed by twisting two skyrmionium lattices shows skyrmionium-bag superlattice at *θ* = 13.2°. The inset shows a skyrmionium bag, where multiple elementary skyrmioniums are surrounded by a bag skyrmionium. **d** Nested-topology superlattices with target skyrmion-skyrmionium mixed topology obtained when *θ* = 27.8°. The inset highlights a target skyrmion composed of multiple radially nested skyrmions with number of *n* ≥ 1. **e** Skyrmion-cluster superlattices in twisted bi-volume C_4_ skyrmion lattices when *θ* = 16.26°. The inset shows a skyrmion cluster texture composed of *n* skyrmions with total skyrmion number of *N*_sk_ = *n*. **f** Skyrmion-bag superlattices in twisted bi-volume C_6_ skyrmion lattices at *θ* = 13.2°. The inset shows a skyrmion bag with *n* elementary skyrmions surrounded by a bag skyrmion with total skyrmion number of *N*_sk_ = *n* − 1

Twisting two such topological lattices with a controlled relative angle gives rise to a freespace moiré field, where the superposition of the individual textures produces entirely new optical structures with novel topologies. The twist couples the intrinsic symmetry of each lattice to an additional spatial degree of freedom, leading to the formation of moiré superlattices with modified periodicity and symmetry. By varying the twist angle and the symmetry of the constituent lattices, we can engineer a broad range of high dimensional topological configurations that do not exist in the individual lattices. This tunability forms the basis of freespace twistronics, allowing direct optical access to moiré physics and emergent topology without the need for material interfaces. As the twist angle increases, the resultant field evolves from regular lattice geometry to complex quasiperiodic and clustered geometries. This continuous transformation marks the onset of high-dimensional topological organization in free space, as shown in Fig. 1. For instance, a C_6_ skyrmionium lattice beam is shown in Fig. 1(a), where the polarization Stokes vectors form a periodic array of skyrmioniums with sixfold rotational symmetry. Each unit cell is a hexagonal skyrmionium composed of two radially nested skyrmions with opposite polarity, giving a total skyrmion number of *N*_sk_ = 0 (see inset). These topological lattices propagate along the *z* axis without diffraction, maintaining both their geometric size and topological structure. However, during propagation, the constituent spatial-frequency components acquire the same longitudinal phase, preserving their relative phase and consequently the local Stokes-vector texture and skyrmion helicity. Notably, in contrast to prior plasmonic topological lattices of Néel-type skyrmions^21,22^, our freespace topological optical lattices offer additional degrees of freedom for controlling the skyrmion helicity, enabling Bloch-, Néel-, and anti-type topological lattices, as shown in Extended Data Fig. E2. Furthermore, by superimposing two volumes of such topological optical lattice fields, we generate moiré structured light fields that host even more complex topologies, as shown in Fig. 1(b). By varying the twist angle, the lattice topology, and the underlying symmetry, a broad family of nontrivial moiré patterns can be realized. These degrees of freedom are optically encoded rather than structurally fixed, making the resulting moiré field digitally programmable and allowing different supertopological configurations to be accessed within the same optical platform.

Depending on the twist angle, the resulting moiré field can form either commensurate (periodic) or incommensurate (aperiodic) geometries. The incommensurate patterns lack translational symmetry, however, the rotational symmetry is preserved. Commensurate patterns are formed when lattice points from both constituent topological lattices coincide, resulting in well-defined periodicity. The commensurability condition can be expressed as^15^

2 $$p{\boldsymbol{a}}+q{\boldsymbol{b}}\to q{\boldsymbol{a}}+p{\boldsymbol{b}}$$

where *p* and *q* are integers that define the coincidence between the primitive lattice vectors ***a*** and ***b*** of the two individual topological lattices.

In the commensurate regime, distinct topological superlattices are formed at specific twist angles. For instance, at *θ* = 13.2° the transverse field forms an optical superlattice composed of repeated supercells of complex skyrmionium bags—a topology not previously reported in any physical system (Fig. 1(c)). Each skyrmionium bag consists of elementary skyrmionium cluster (e.g., *n* = 7) surrounded by a larger bag skyrmionium of opposite polarity.

Controlled variation in the twist angle leads to a variety of new supertopologies, including optical nested-topology superlattices (see Extended Data Fig. E3). Fig. 1(d) shows a twisted bi-volume C_6_ skyrmionium lattice field at *θ* = 27.8^◦^, where a nondiffracting nested-topological superlattice is formed in free space, where the skyrmioniums at the periphery are shared with neighboring super unit cells. Our freespace optical nested-topology superlattice show a new class of higher-order mixed topology, where the center hosts a target skyrmion composed of multiple radially nested skyrmions (*n* ≥ 1) with a total skyrmion number |*N*_sk_| = [1 − ( − 1)^*n*^]*/*2, surrounded by a set of skyrmioniums. Furthermore, for specific twist angle, freespace supertopologies lead to a quasicrystalline geometry similar to those recently reported in plasmonic systems^32–34^. Quasicrystals are ordered but aperiodic systems that exhibit rotational symmetry while lacking translational symmetry, yet can host nontrivial topological textures such as the skyrmion–meron mixed states observed in plasmonic quasicrystals^33^. For *θ* = 30° a new class of higher-order mixed topology can be obtained and termed as *supertopological quasicrystals* (see Supplementary Material S1).

Furthermore, the individual C_6_ skyrmionium lattices can be replaced with other topological lattices to obtain more diverse topologies. For instance, when C_4_ skyrmion lattices are twisted, the moiré field at *θ* = 16.26° exhibits a new type of topology not previously reported—the skyrmion-cluster superlattice (Fig. 1(e)). The inset shows a skyrmion-cluster supercell that hosts *n* skyrmions of identical topology with a total skyrmion number of |*N*_sk_| = *n*. Similarly, for C_6_ skyrmion lattices, the moiré field at *θ* = 13.2° forms skyrmion-bag superlattices (Fig. 1(f)). In a skyrmion bag, multiple elementary skyrmions (*n* = 7) are enclosed by a larger skyrmion bag of opposite polarity, giving a total skyrmion number of |*N*_sk_| = *n* − 1 (see inset). Notably, the skyrmion bag topology was previously proposed in liquid crystals and magnetic systems^36^, and more recently observed in surface plasmon polaritons on metal surfaces^15^. In contrast, the skyrmion bags demonstrated here are realized in freespace light fields that can propagate over extended nondiffracting distances while preserving their topology.

In addition to the twist angle and constituent lattice symmetry, the center of rotation also provides another degree of freedom for controlling the generated supertopologies. Off-center twisting about different symmetry points changes the relative transverse registration of the constituent topological lattices and consequently modifies the resulting supertopology (see Supplementary Material S2).

### Moiré supertopologies

**Fig. 2:**
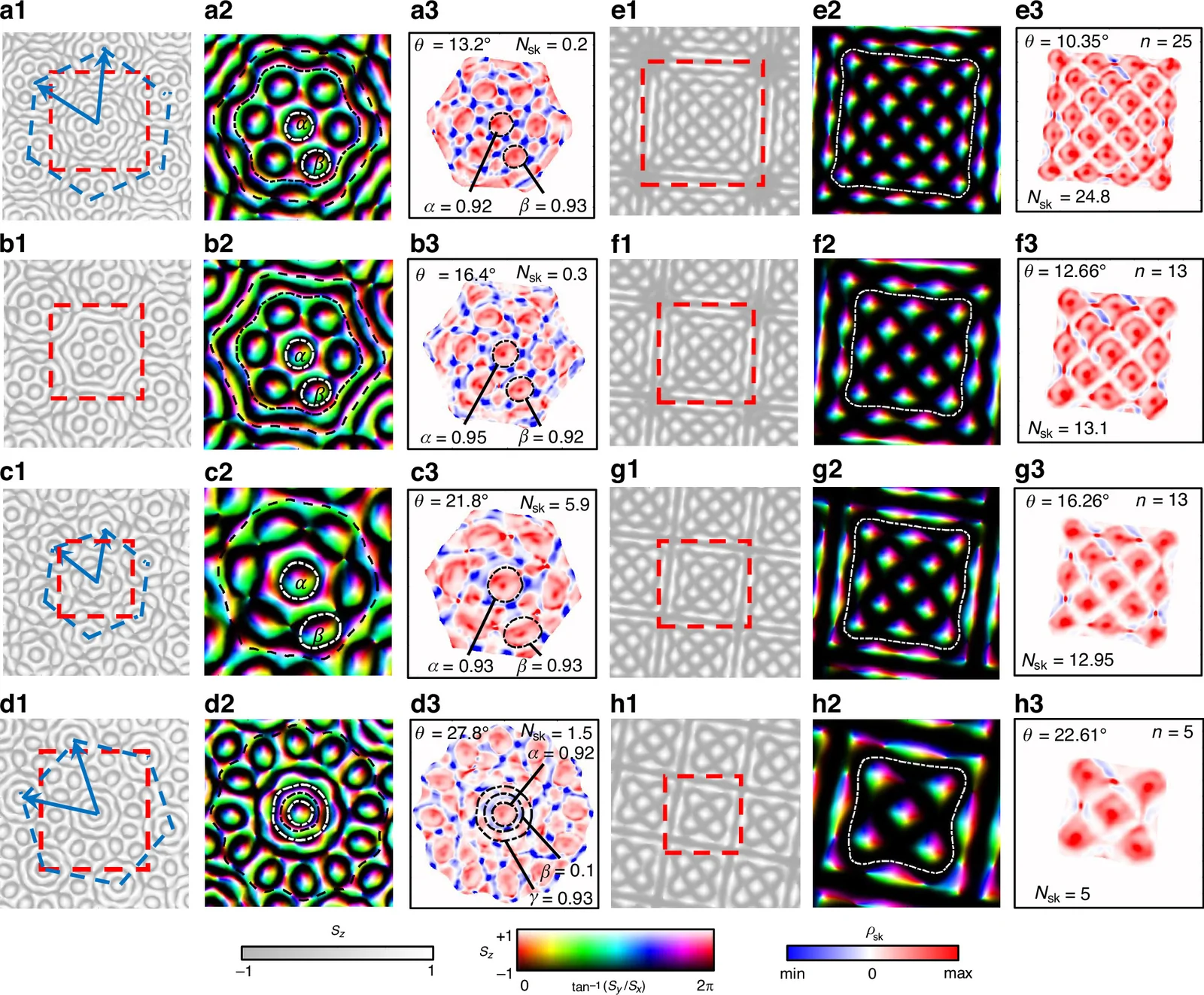
Experimental results of optical supertopologies. Long-range distributions of *S*_*z*_, spin textures of featured topological structures (in red-dashed line boxes of long-range patterns correspondingly), and corresponding skyrmion density distributions for twisted C_6_ skyrmionium lattices obtaining (**a1**–**a3**) skyrmionium-bag superlattices at *θ* = 13.2° (blue arrows mark the long-range periodic unit vectors of supercell), (**b1**–**b3**) skyrmionium bag at *θ* = 16.4°, (**c1**–**c3**) skyrmion-cluster superlattices at *θ* = 21.8°, (**d1-d3**) nested-topology superlattice at *θ* = 27.8° and for twisted C_4_ skyrmion lattices obtaining skyrmion-cluster superlattices for different baby skyrmion numbers in supercell of (**e1**–**e3**) *n* = 25 at *θ* = 10.35°, (**f1-f3**) *n* = 13 at *θ* = 12.66°, (**g1**–**g3**) *n* = 13 at *θ* = 16.26°, (**h1**–**h3**) *n* = 5 at *θ* = 22.61°. The experimental skyrmion numbers *N*_sk_ of featured supertopologies, marked by big dashed lines in (**a2**–**h2**), are shown in (**a3**–**h3**), respectively. The experimental *N*_sk_ values of selected protected baby skyrmions (“*α*”,“*β*”,“*γ*”,) marked by black dashed lines are shown in (**a3**–**d3**), correspondingly

To demonstrate the proof-of-concept, we experimentally realize the freespace supertopologies using a spatial light modulator (SLM) (see Supplementary Material S3). The constituent lattice fields and their relative twist angles are encoded through computer-generated holograms, allowing the supertopological configuration to be digitally reprogrammed within the same experimental platform. We first present the results for twisted C_6_-based skyrmionium lattices at *θ* = 13.2°, where superlattices of skyrmionium bags act as supercells arranged in long-range C_6_ symmetry. Fig. 2(a1) shows the measured long-range order in the out-of-plane Stokes vector *S*_*z*_, where the arrows indicate the unit vectors defining the hexagonal supercells formed by the skyrmionium bags. The polarization texture of the central skyrmionium bag is shown in Fig. 2(a2), where the black dash–dot line marks the boundary of the skyrmionium cluster formed by seven individual skyrmioniums. This cluster is enclosed within a larger skyrmionium bag of opposite polarity, whose boundary is outlined by the dashed black curve. The boundaries are identified using a computational Snake algorithm (see Methods). Fig. 2(a3) shows the measured skyrmion density for the central skyrmionium bag. As evident, the elementary skyrmioniums within the cluster share the same vorticity as the surrounding bag skyrmionium but with opposite polarity. The measured skyrmion numbers of the entire skyrmionium bag and its inner skyrmion cluster are both close to zero, whereas each baby skyrmion embedded in the cluster exhibits a near-unit topology (*N*_sk_ > 0.9).

As the twist angle increases to *θ* = 16.4°, the overall long range C_6_ superlattice exhibits an increased spatial period. Figure 2(b1) shows that the central skyrmionium bag remains intact, while the surrounding region forms a larger-period superlattice. The corresponding polarization and skyrmion density maps (Figs. 2(b2,b3)) confirm that the core topology of the central skyrmionium bag is preserved despite the change in superlattice periodicity. Further increasing the twist angle to *θ* = 21.8° leads to a topological transition into the skyrmion-cluster superlattice, as shown in Fig. 2(c1). This topological texture can be named as *skyrmion-skyrmionium bag*, where the central skyrmionium is surrounded by multiple skyrmions, all enclosed within an outer bag skyrmion. This is a novel topology of skyrmion bag not realized in plasmonic before. In this configuration, the supercells adopt a petal-like arrangement consisting of multiple skyrmion clusters surrounding a central skyrmionium. The polarization and skyrmion number density plots (Figs. 2(c2,c3)) show that the central skyrmionium and its protected skyrmion exhibit higher structural fidelity compared to the six surrounding petals.

At *θ* = 27.8°, a nested-topology superlattice emerges, where the basic central structure is a multi-π nested target skyrmion surrounded with multiple skyrmionium tiling the whole space, with global translational symmetry (Fig. 2(d1)). The first three nested skyrmions at the center with measured skyrmion numbers close to *±*1 were analyzed in Figs. 2(d2,d3). Furthermore, we generate diverse moiré supertopologies by performing freespace twistronics on C_4_-based skyrmion lattices, resulting in skyrmion-cluster superlattices that contain different numbers of skyrmions per supercell at different twist angles. At *θ* = 10.35°, the moiré superlattice supercell contains 25 skyrmions (Figs. 2(e1–e3)). When the twist angle is increased to *θ* = 12.66°, the structure transforms into a 13-skyrmion supercell (Figs. 2(f1–f3)), which remains stable up to *θ* = 16.26° (Figs. 2(g1–g3)). Further increase to *θ* = 22.61° results in a 5-skyrmion supercell (Figs. 2(h1–h3)). Simulation results corresponding to Fig. 2 are presented in Extended Data Fig. E4. For the C_4_-based skyrmion lattices, there is no one-to-one mapping between the vortices and Gaussians in the LCP and RCP components, leading to intensity nulls or electromagnetic dark spots within the lattice. When such lattices are twisted, these intensity nulls persist in the resulting moiré superlattices, which can introduce small inaccuracies in the measurement of total skyrmion number. To ensure accurate measurements, a small correction term *δ* is added to the LCP component. The correction term *δ* preserves the superlattice topology but removes intensity nulls and singularities from Stokes vector *S*_*z*_, yielding an accurate calculation of skyrmion number (see Supplementary Material S4). Furthermore, freespace twistronics is also performed on C_3_-based meron lattices, yielding complex meron cluster supertopologies (see Supplementary Material S5). Figs. 2(a–d) show the topological lattice obtained when the LCP vortex lattice carries unit topological charge. For the C_6_ case, however, a vortex lattice with topological charge 2 can also be generated. This enables a mixed-topology lattice in which skyrmions with skyrmion number 2 can be obtained (see Supplementary Material S6).

**Fig. 3:**
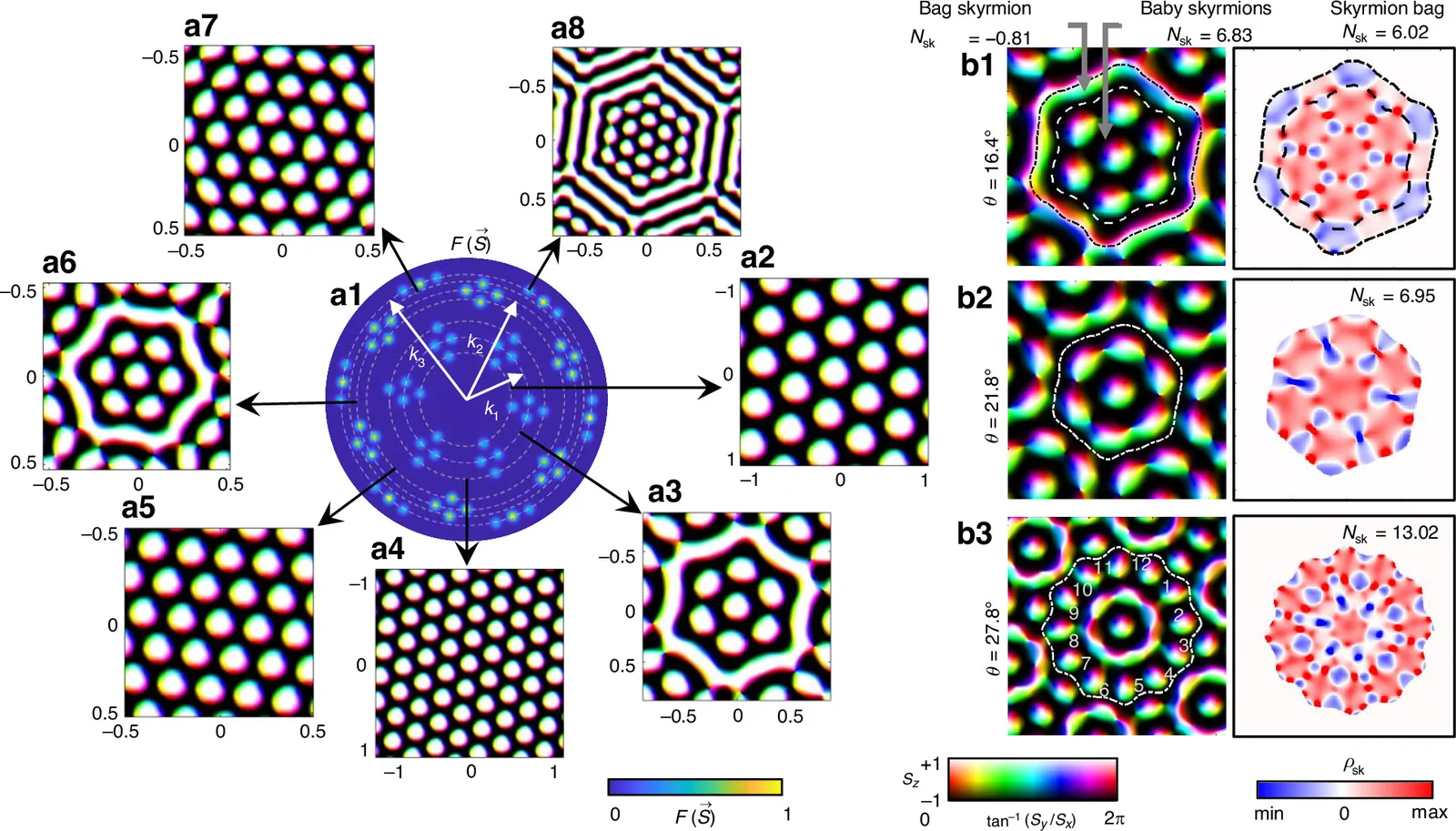
Hierarchical supertopology decomposition. **a1** Theoretical spatial Fourier spectrum of Stokes vector of a skyrmionium-bag at *θ* = 16.4°, where the distribution is located at a set of concentric circles in wavenumber-space, and **a2**–**a8** the corresponding hierarchical real-space textures as skyrmion lattices and skyrmion-bag superlattices. The white arrows in (**a1**) mark the three circles of wavenumber radii *k*_1_, *k*_2_, and *k*_3_ for the cases of skyrmion bag generation. **b1**–**b3** Experimental results of skyrmion-bag, skyrmion-cluster superlattices, and nested-topology, with measured textures and skyrmion density distributions, at twist angles 16.4°, 21.8°, and 27.8°, respectively; measured skyrmion numbers of selected topological textures in dashed lines are marked. Unit of coordinates: mm

Moreover, it has been shown that due to periodicity in the Stokes vector, the topological lattices can be decomposed in sub-lattices of lower topologies. For example, a C_6_ skyrmionium lattice can be decomposed in three different C_6_ skyrmion sub-lattices of different periodicity. Similarly, a C_4_ skyrmion lattice can be decomposed in two C_4_ meron sub-lattices^25^. Therefore, in addition to the supertopologies discussed above, we can also achieve skyrmion bags, skyrmion-petal superlattices, lower-order topological quasicrystals, and so on. To observe freespace skyrmion-bag superlattices, we need the optical lattice decomposition method^25^. Similar to the Stokes vector of individual C_6_ skyrmionium lattices, the Stokes vector of twisted superlattice also exhibits periodicity, leading to various spatial frequency components. Therefore, by selective spatial decomposition, lower order topologies can be obtained. Fig. 3(a1) shows the spatial Fourier spectrum of the Stokes vector (Fig. 2(b1)) corresponding to skyrmionium-bag superlattice obtained by twisting two elementary skyrmionium lattices at an angle of *θ* = 16.4°. The Fourier spectrum can be divided into seven sets of spatial frequency components (shown by white dashed circles in Fig. 3(a1)). Figures 3(a2-a8) show 7 real-space textures which are obtained from the seven different spatial frequency circles. These textures resemble C_6_ skyrmion lattices, as well as skyrmion bags in bilayer and trilayer moiré skyrmion superlattices. Spatial frequencies consisting of only six spots result in C_6_ skyrmion lattices in real space with different lattice vectors (Figs. 3(a2-a8)). However, sets of spatial frequencies consisting of more than six spots, i.e. *k*_1_ and *k*_2_ with twelve and *k*_3_ with eighteen spots result in higher order supertopologies. Note, *k*_1_ and *k*_2_ result in skyrmion bag corresponding consisting of seven elementary skyrmions (Figs. 3(a3,a6)) representing bilayer twisting of skyrmion lattices, whereas, *k*_3_ generates a bigger skyrmion bag with eighteen elementary skyrmions (Fig. 3(a8)) which represents trilayer twisting of skyrmion lattices. Figure 3(b1) shows the experimental results of the skyrmion bag, where the *n* = 7 baby skyrmions are inside with measured *N*_sk_ = 6.83 surrounded with a bag skyrmion of opposite polarity with *N*_sk_ = *−*0.81, thus, the total skyrmion number is *N*_sk_ = 6.83 − 0.81 = 6.02 and its absolute value is close to the ideal *n* − 1 = 6 (Fig. 3(b1)). If we do same decomposition to moiré textures at *θ* = 21.8°, we can obtain skyrmion-cluster superlattice, where the supercell is petal-like cluster of 7 skyrmions (Fig. 3(b2)). At *θ* = 27.8°, the decomposed moiré texture shows a lower-order nested-topology superlattices, where the basic structure is a 3π target skyrmion surrounded with 12 skyrmions, thus the total skyrmion number is close to |*N*_sk_| = 13 (Fig. 3(b3)). Complete hierarchical supertopology decomposition for other twist angles is shown in Extended Data Fig. E5. Furthermore, hierarchical supertopology decomposition for twisted *C*_4_ skyrmion lattices and *C*_3_ meron lattices reveals sub-lattices of lower topologies such as meron lattices and clusters (see Supplementary Material S7–S8). We can apply such decomposition to other moiré supertopologies to access more kinds of topologies. The relative amplitude *µ* for the constituent polarization components provides an additional degree of freedom for modifying the supertopological texture and its hierarchical composition. Its influence on the real-space texture and the decomposed *k* components is further investigated in Supplementary Material S9.

### Topological robustness

**Fig. 4:**
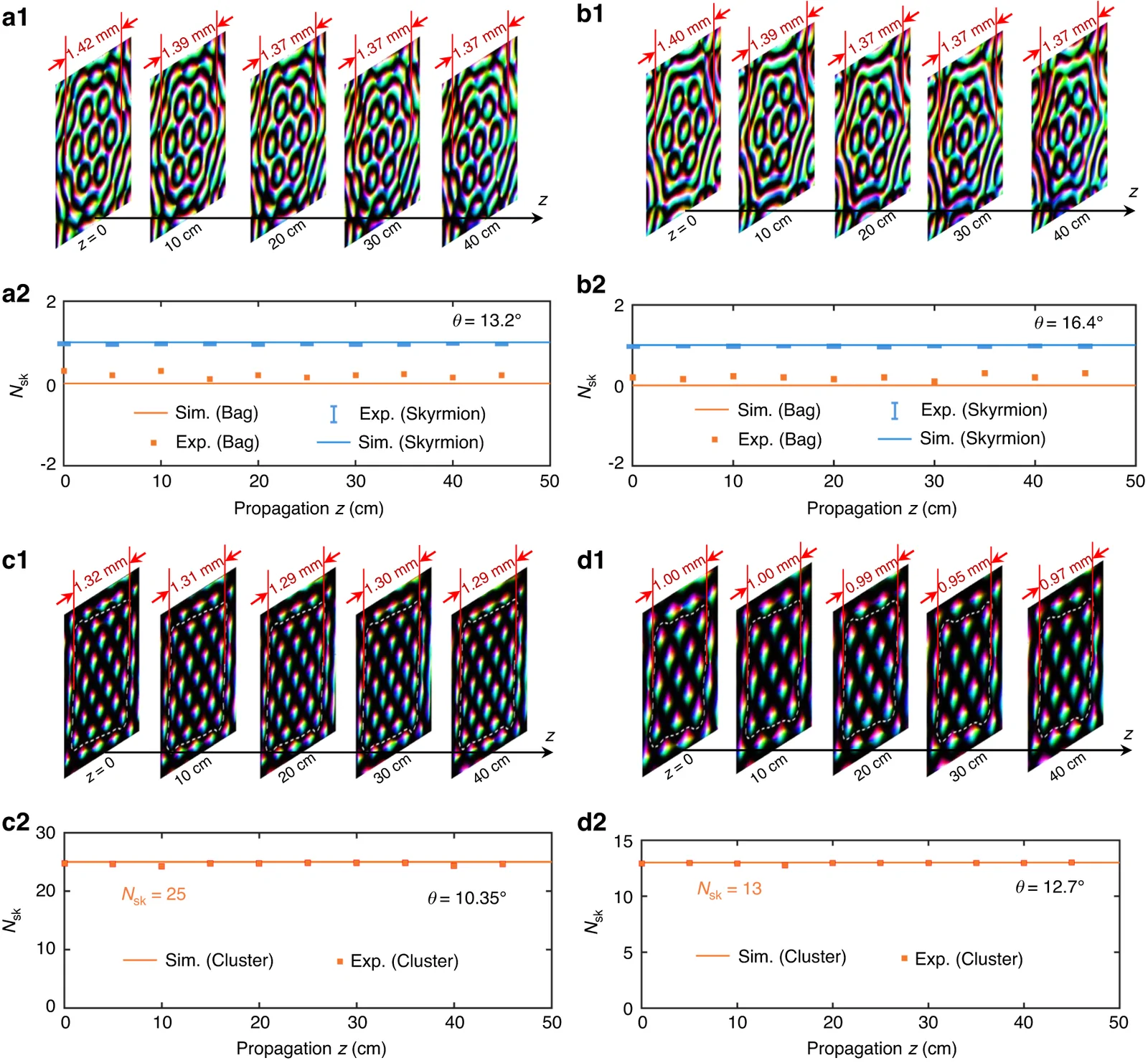
Nondiffracting topologically stable propagation. **a1**–**d1** Experimental results of the propagation-dependent supertopological textures for two skyrmionium bags at *θ* = 13.2° and 16.4° and two skyrmion clusters at *θ* = 10.35° and 12.7°, respectively, from *z* = 0 to 40 cm. The featured side is marked correspondingly in each texture pattern. **a2**, **b2** The simulated and experimental skyrmion numbers for the skyrmionium bags (*n* = 7) and the protected baby skyrmions (in which the error bars evaluate the numerical difference for the 7 baby skyrmions), corresponding to (**a1**, **b1**). **c2**, **d2** The simulated and experimental skyrmion numbers for the skyrmion clusters (*n* = 25 and *n* = 13), corresponding to (**c1**, **d1**). Note, the error bars in (**a2**, **b2**) represent the variation in the measured skyrmion numbers among the seven individual baby skyrmions within the skyrmionium bag. Whereas, in (**c2**, **d2**), each data point represents the total skyrmion number integrated over the skyrmion clusters, and therefore no error bars are shown

The supertopologies are generated by twisting the light fields of two elementary topological lattices. Therefore, they exhibit the propagation properties of elementary lattices. To understand this, consider an elementary C_6_ skyrmionium lattice, with Fourier spectrum of six discrete spatial-frequency components uniformly distributed on a ring of constant radial spatial frequency. In an ideal *N*-wave interference model, the point sources are located at the same radial spatial frequency and therefore possess the same longitudinal propagation constant, resulting in nondiffracting lattices. Experimentally, these point sources are approximated by finite-width Gaussian spots in the Fourier plane. Therefore, the central spatial-frequency components satisfy the same condition, while the finite spectral width introduces a small spread of longitudinal propagation constants and limits the nondiffracting range. The finite transverse extent of the fields and optical aperture impose additional limits on the nondiffraction property. Therefore, as a proof-of-concept, we experimentally demonstrate the nondiffracting characteristics of the supertopologies over 50 cm. In contrast to conventional skyrmionic or bimeronic beams where they are divergent but topologically invariant upon propagation^37^. Here the topological features and geometric shape and size the supertopologies all do not change at all. Figures 4(a,b) show experimentally measured skyrmion bag textures upon propagation through 50 cm, in twisted C_6_ skyrmionium lattices at *θ* = 13.2° and 16.4°. In both cases, the geometric sizes and topological features are invariant upon propagation; see details in Extended Data Figs. E6-E7. Figures 4(a2,b2) are corresponding simulated and experimental skyrmion numbers for the skyrmionium bags (*n* = 7) and the protected baby skyrmions in which the error bars evaluate the numerical difference for the 7 baby skyrmions. The results prove that supertopology is resilient to both dynamic propagation and twisted angle deviations.

Figs. 4(c,d) show experimentally measured nondiffractive propagation-dependent geometry-independent skyrmion clusters in twisted C_4_ skyrmion lattices at *θ* = 10.35° and 12.7° for *n* = 25 and *n* = 13, respectively. The measured skyrmion numbers agree very well with the theoretical prediction; see Figs. 4(c1,d1). Please see details in Extended Data Figs E6-E7. The propagation invariance discussed above corresponds to the coaxial propagation. We further investigate non-coaxial twisting by introducing a transverse displacement of the spatial-frequency distribution of one lattice, thereby tilting its propagation axis relative to the other. In this case, the resulting propagation-dependent relative phase leads to a gradual deformation of the supertopological texture along *z* (see Supplementary Material S10).

Hereinafter, we show the nondiffractive supertopologies are even strongly resilient to obstacles. Notably, the topological protection of skyrmions was previously studied^38,39^, however, these studies focused on complex transparent and scattering media, and no similar studies on topological lattices have been reported. The blocking by solid obstacles can introduce a stronger perturbation in contrast to complex media. The optical mode revival after encountering obstacles in propagation path is usually called self-healing effect^40^. The topological self-healing of skyrmionic beam was also studied recently^41^, but again, not for topological lattice.

**Fig. 5:**
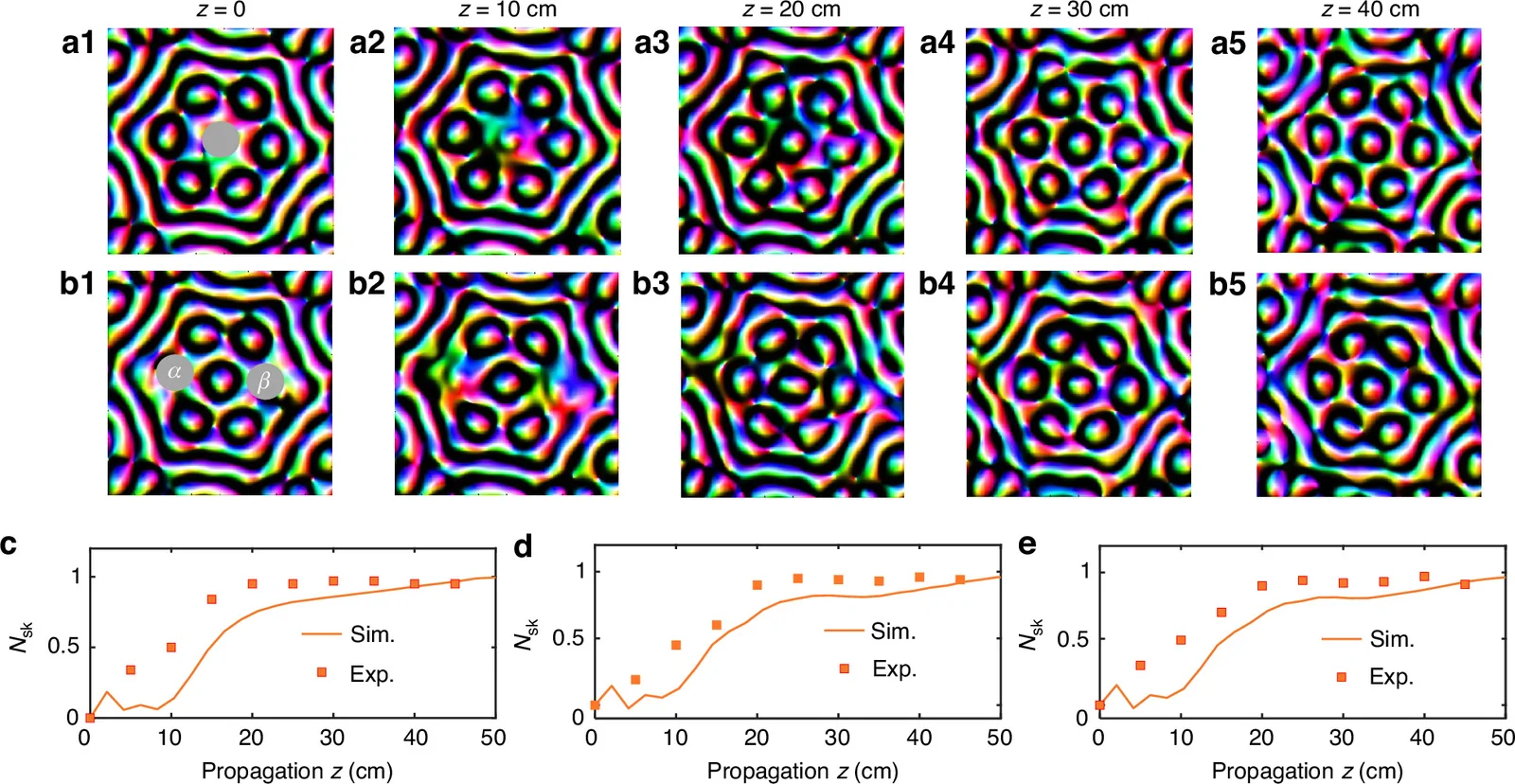
Self-healing topologies against obstacles. Experimental results of skyrmionium bag propagation passing through obstacles for (**a1-a5**) a disk, marked in gray, blocking the center skyrmionium and (**b1-b5**) two disks blocking two skyrmioniums in bag, at *z* = 0. **c**–**e** The simulated and experimental skyrmion number versus propagation for the baby skyrmion protected in the blocked skyrmionium in (**a1**) and that two in the two blocked skyrmioniums (“*α*” and “*β*”) in (**b1**), respectively

When a finite portion of such a field is obstructed, the remaining wavevector components continue to propagate and interfere, gradually reconstructing the locally obstructed field. Therefore, the self-healing observed here primarily originates from the angular-spectrum structure of the nondiffracting optical lattice rather than from the polarization topology itself. Figures 5(a,b) show our experimental results of skyrmionium bag propagation passing through obstacles consisting of one disk (a) and two disks (b), blocking one and two skyrmioniums in the bag, respectively. The simulated and experimental skyrmion number versus propagation for the baby skyrmion protected in the blocked skyrmionium and that two in the two blocked skyrmioniums (“*α*” and “*β*”) are shown in Figs. 5(c-e), respectively (Please see simulation results in Extended Data Fig. E8).

It is evident that supertopologies exhibit self-healing behavior against perturbations, as the skyrmion number gradually recovers to its initial value during propagation. The topological resilience is complementary to the wave-optical self-healing mechanism. Here, the topology is encoded in the normalized Stokes-vector field, where mapping onto the Poincaré sphere is characterized by the skyrmion number *N*_sk_. Under continuous and non-singular perturbations, this topological degree remains invariant^38,39^. Therefore, polarization provides the vector degree of freedom carrying the skyrmion topology, rather than being the origin of self-healing. After a local obstruction, the Stokes texture may be strongly distorted. However, as the optical field reconstructs during propagation, the Stokes-vector field is gradually reconstructed along with its associated skyrmion topology.

However, the observed robustness is also constrained by the strength and nature of the perturbation. The self-healing distance and fidelity depend on the size and position of the obstruction and on the remaining angular-spectrum components, while the finite aperture of a practical optical system limits the nondiffracting propagation range.

## Discussion

We proposed the concept of optical moiré supertopologies generated from a controlled interlayer twist between two optical topological lattices of free space. Many novel topologies, skyrmionium bags, skyrmion-bag superlattices, supertopological quasicrystals, etc., not accessible in any physical system before, now, can be simply generated in twisted bi-volume moiré structured light. In this work, we only considered superposition of two layers. It can be extended to moiré-of-moiré scheme as recently emerging trilayer lattices^3,42^ and twisted multilayer^6^ for opening unlimited exploration of more nontrivial topologies. In the experiment, the digital programmability of the constituent lattices and their relative twist angle provides a flexible platform for exploring transitions among complex topological states.

Breaking 2D limit of prior material dependent “twistronics”, we open new dimensions and more degrees of freedom for 3D even 4D space-time twisting in topological lattices of light. In this work, we mainly considered coaxial twisting, while for emerging 3D freespace topological optical field, e.g., the space-time hopfion crystals^27^, we can open 3D twisting of multiple topological lattices along arbitrary axial orientations for potential higher-dimensional space-time supercrystals. Although the present work focuses on topological textures encoded in the Stokes vector, the concept of freespace twistronics is not restricted to polarization degrees of freedom of light. More generally, periodic topological textures defined by other optical vectors, including the optical spin^20,22,31,34^, electromagnetic field^15,21,34^, spin–momentum flows^43^ and energy-flow vectors^44^, could serve as constituent lattices for corresponding spin-, field-, or flow-based twistronic systems.

In contrast to any prior model of moiré electronics and photonics relying on responses of materials, our freespace moiré supertopological light can propagate so as to interact with matter, potentially transfer optical topologies to matter, as a new methodology to excite polaritonic topologies^45^, atomic topologies^46^, for atom trapping^47,48^, and structured light directly writing topological solitons of matter. The moiré supertopologies can be utilized as an excellent platform to investigate more properties of structured light such as energy localization^11^ and superoscillation^49^.

Optical supertopologies are in high demand for next-generation information carriers to revolutionize our optical communication network^50^. Recent demonstrations of skyrmion-based encoding further highlight its potential as robust and broadband information carriers^51,52^. The digital programmability of distinct supertopological textures provides a larger space for information encoding, where different lattice symmetries, twist configurations, and topological states may serve as addressable channels. Combined with nondiffracting propagation and resilience to perturbations, such reconfigurable states may offer potential applications in robust high-dimensional optical information transfer and encryption.

Supertopologies were first generated in light waves here, which will certainly motivate the exploration of supertopologies in other waves, e.g., sound waves^53^ and water waves^54^, as well as the exploration of supertopologies in matter, i.e., liquid crystals^19,36^, chiral magnets^29^ and beyond. In general, wave systems supporting periodic topological textures and coherent superposition may provide promising platforms for extending the concept of twistronic supertopologies beyond optics.

## Methods

### Characterization of skyrmion and its boundary

The topological properties of the optical skyrmions can be characterized by the skyrmion number, which is defined as^28^

$${N}_{\mathrm{sk}}=\frac{1}{4\pi }\mathop{\iint }\limits_{\sigma }{\boldsymbol{S}}\cdot \left(\frac{\partial {\boldsymbol{S}}}{\partial x}\times \frac{\partial {\boldsymbol{S}}}{\partial y}\right){\rm{d}}x{\rm{d}}y$$

where ${\boldsymbol{S}}$(*x,y*) represents the Stokes vectors to construct a skyrmion and *σ* denotes the skyrmion area. For the experimentally measured Stokes fields, we applied linear interpolation followed by a Gaussian filter as part of the post-processing procedure to suppress noise and enhance the accuracy of the skyrmion number calculation. Besides, due to the different size of the skyrmionic Stokes fields, careful area selection was crucial.

Ideally, the Stokes skyrmion is well-defined with clear circular symmetry, allowing the topological charge to be determined by integrating over a circular contour. However, turbulence disrupts this symmetry and a circular integral no longer gives us the correct topological charge. Therefore, it is necessary to accurately define an adaptive contour that accurately captures the topological charge. Here, we applied the Active Contour Model (ACM), otherwise known as Snakes^55^. Snakes are an unparalleled tool widely employed in computer vision, primarily used to outline an object’s boundary in a noisy two-dimensional environment. They are adaptive, energy-minimizing contours that evolve iteratively under the influence of internal and external forces, denoted by *E*_int_ and *E*_ext_, respectively. Mathematically, the Snake’s energy function is given by:

$${E}_{{\rm{snake}}}={\int^{1}_{0}}({E}_{\mathrm{int}}+{E}_{{\rm{ext}}}){\rm{d}}s$$

where ${\rm{d}}s$ represents the differential element of the curve parameter. The optimization of the snake function is based on the concept of gradient descent. Starting from an initial contour, the algorithm iteratively minimizes the Snake function’s energy, moving in the direction of the negative gradient at each step. This process continues until the local minima are found, at which point the contour outlines the boundary of the image.

The initial contour is selected using the known geometry of the underlying lattice and its *S*_*z*_ distribution. For irregular supertopologies, including skyrmion clusters and skyrmionium bags, an approximate contour enclosing the relevant *S*_*z*_ extrema is used as the initial input to the Active Contour Model. The initial contour follows the symmetry of the topological lattice. Therefore, a square contour is chosen for C_4_-based supertopologies whereas a hexagonal contour is chosen for C_6_-based supertopologies. For individual skyrmions, a circular or square initial contour is used according to the local geometry of the texture. The contour subsequently evolves through the Snake algorithm to obtain the boundary used for integration of the skyrmion-number density.

## Supplementary information

Supplementary Materials for Freespace "twistronics": twisted structured light for optical supertopologies

Extended Data figures

## Data Availability

The data that support the findings of this study can be accessed from NTU Research Data Storage Finder at https://doi.org/10.21979/N9/AJB6FD.
