# Supplementary material for: Freespace “twistronics”: moiré structured light for optical supertopologies
Source: Light Sci Appl. 2026 Nov 6;15:396. doi: 10.1038/s41377-026-02533-w (PMC13740294; doi:10.1038/s41377-026-02533-w)
Supplement: Supplementary file 1 [file 41377_2026_2533_MOESM2_ESM.pdf]

Extended Date Figures

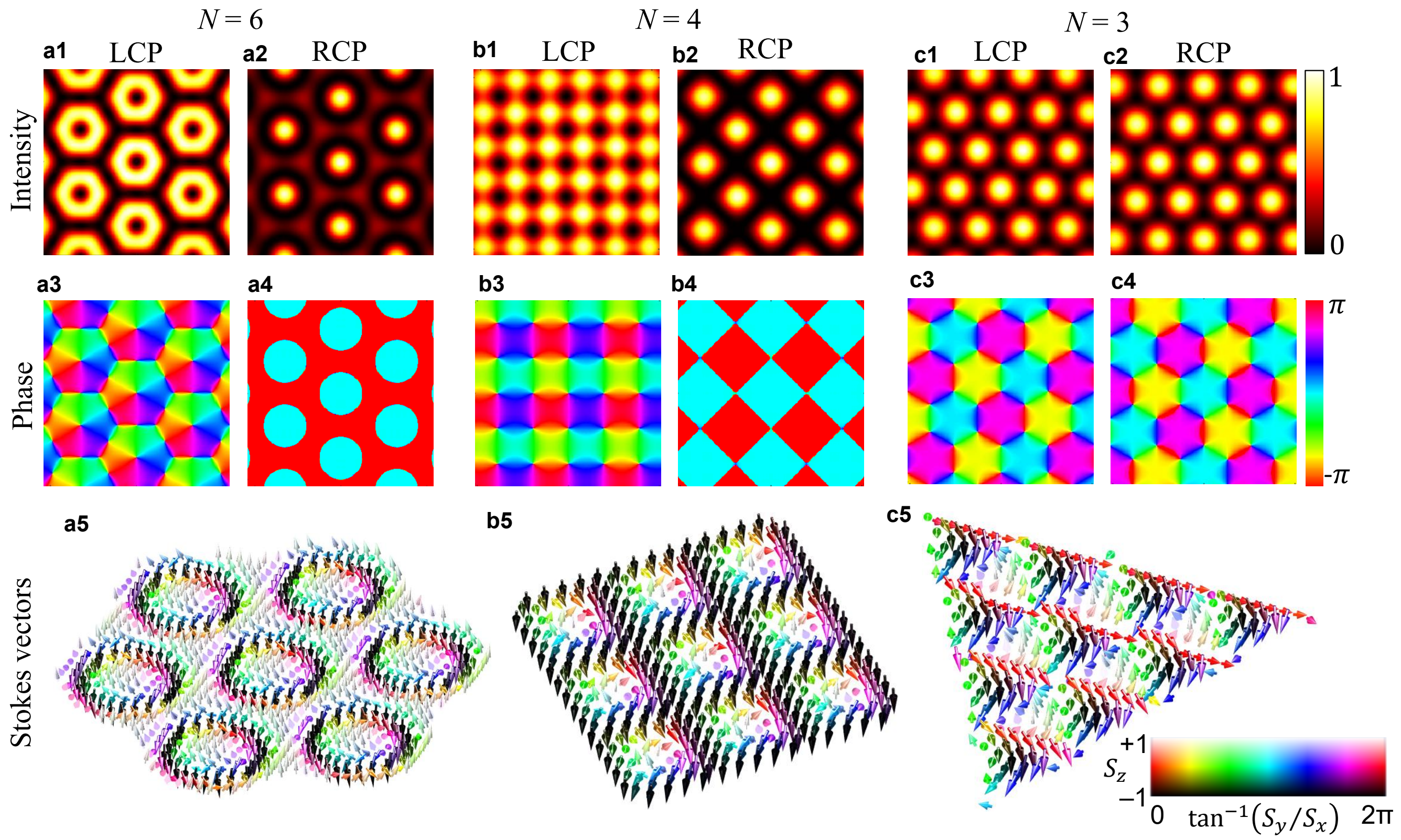

FIG. E1: **Basic topological lattices.** (a1-a2) Intensity and (a3-a4) phase distribution of LCP and RCP whereas, (a5) Stokes vector topological texture for C_6 skyrmionium lattices. (b1-b2) Intensity and (b3-b4) phase distribution of LCP and RCP whereas, (b5) Stokes vector topological texture for C_4 skyrmion lattices. (c1-c2) Intensity and (c3-c4) phase distribution of LCP and RCP whereas, (c5) Stokes vector topological texture for C_3 meron lattices.

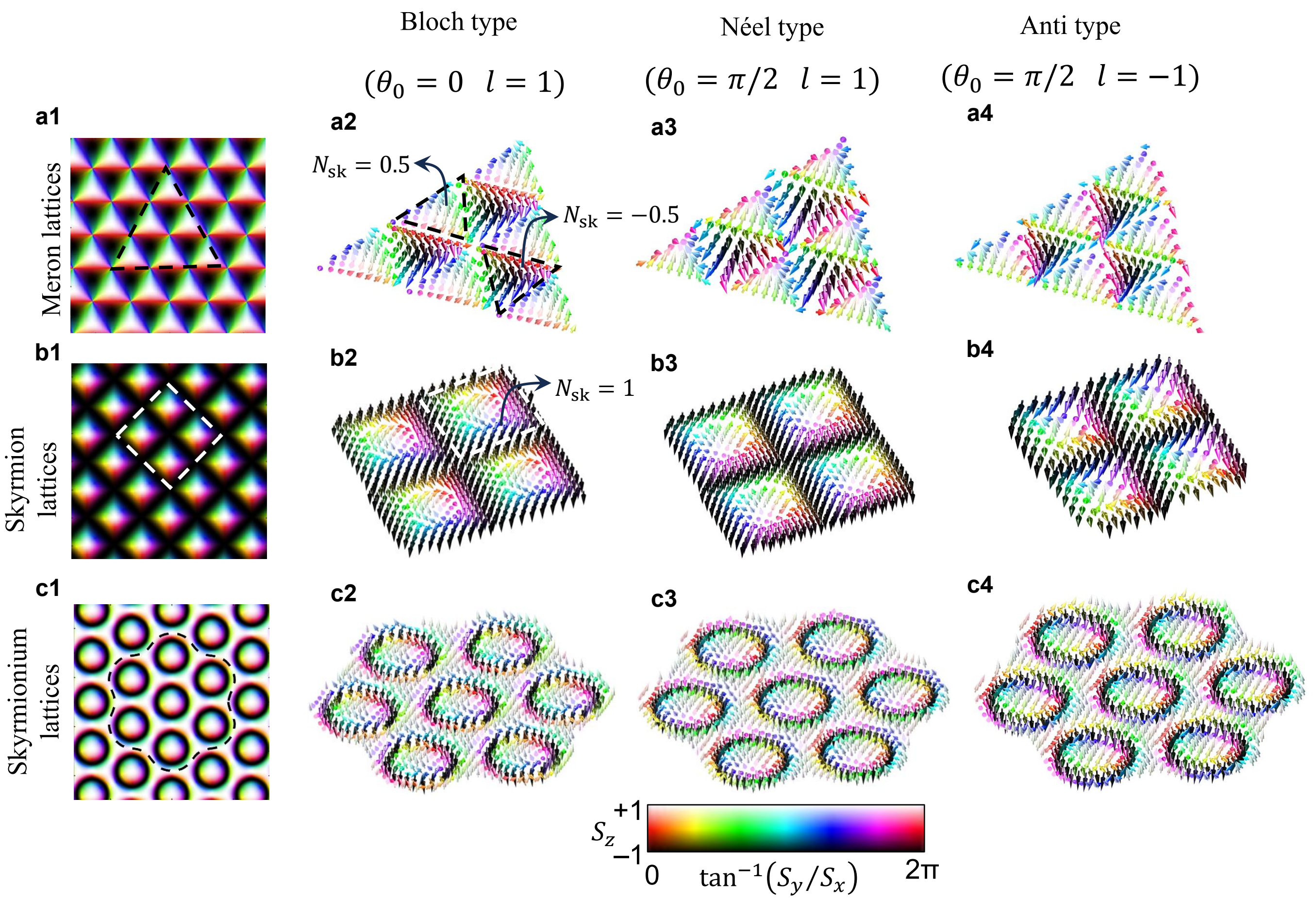

FIG. E2: **Diverse topological lattices.** (a1-a4) Polarizarion texture and Stokes vectors for Bloch, Nèel and anti type C_3 meron lattices. (b1-b4) Polarizarion texture and Stokes vectors for Bloch, Nèel and anti type C_4 skyrmion lattices. (c1-c4) Polarizarion texture and Stokes vectors for Bloch, Nèel and anti type C_6 skyrmionium lattices.

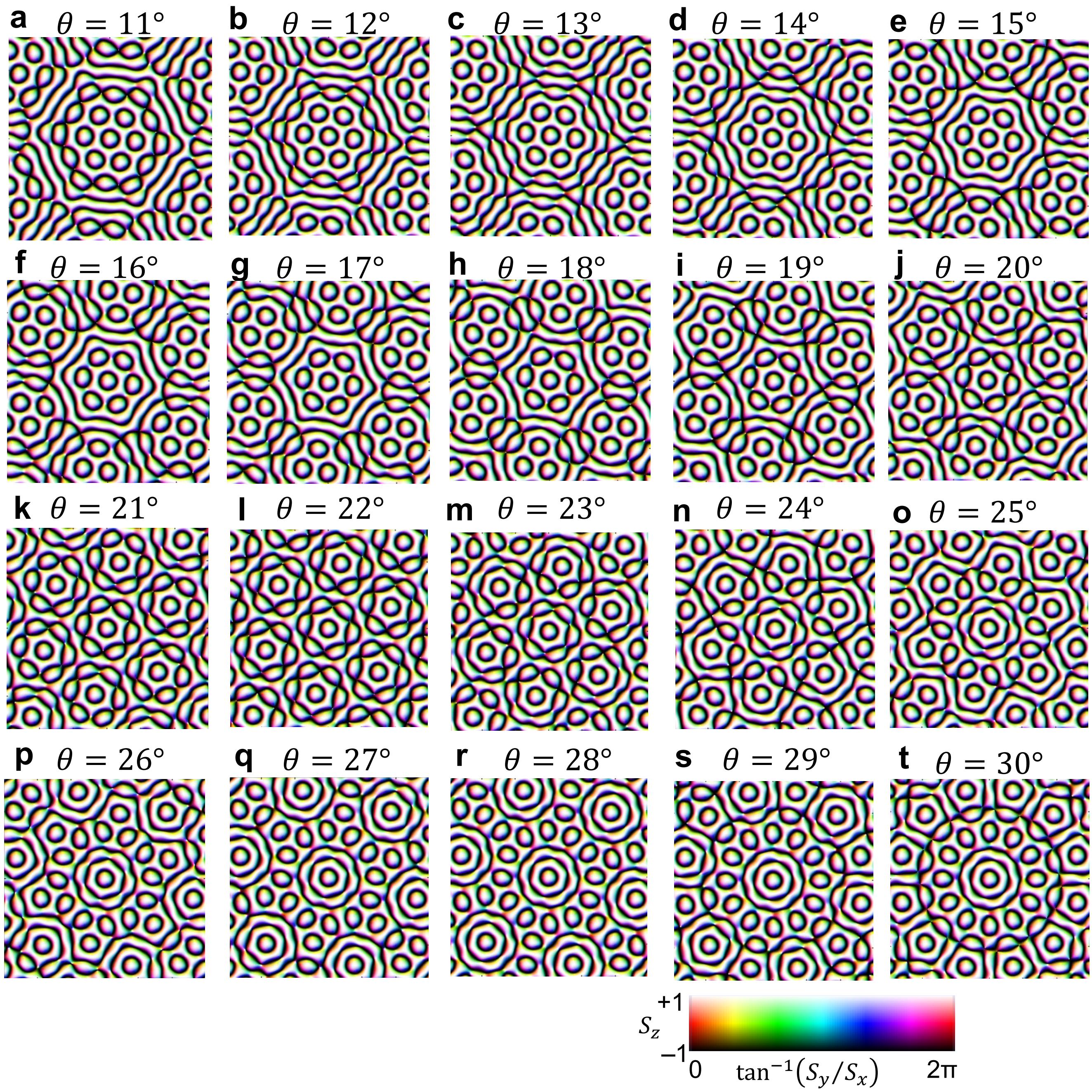

FIG. E3: **Different supertopologies for various twist angles.** (a-t) Polarization texture for supertopologies obtained for twist angles $\theta = 11^\circ - 30^\circ$ for C_6 skyrmionium lattices.

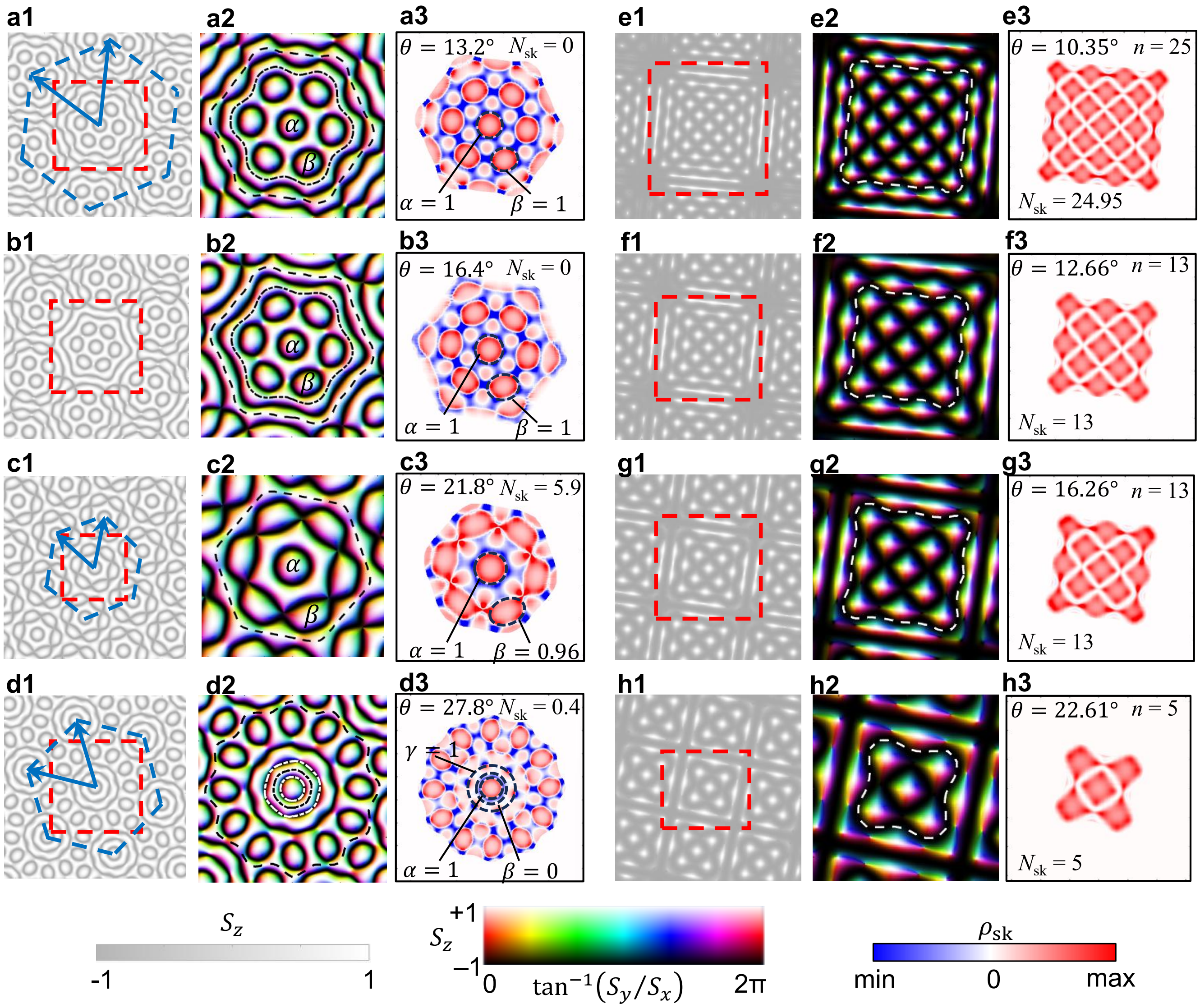

FIG. E4: **Simulation results for various supertopologies.** Long-range distributions of S_z, spin textures of featured topological structures (in red-dashed line boxes of long-range patterns correspondingly), and corresponding skyrmion density distributions for twisted C_6 skyrmionium lattices obtaining (a1-a3) skyrmionium-bag superlattices at $\theta = 13.2^\circ$ (blue arrows mark the long-range periodic unit vectors of supercell), (b1-b3) skyrmionium bag at $\theta = 16.4^\circ$, (c1-c3) skyrmion-cluster superlattices at $\theta = 21.8^\circ$, (d1-d3) nested-topology superlattice at $\theta = 27.8^\circ$; and for twisted C_4 skyrmion lattices obtaining skyrmion-cluster superlattices for different baby skyrmion numbers in supercell of (e1-e3) $n = 25$ at $\theta = 10.35^\circ$, (f1-f3) $n = 13$ at $\theta = 12.66^\circ$, (g1-g3) $n = 13$ at $\theta = 16.26^\circ$, (h1-h3) $n = 5$ at $\theta = 22.61^\circ$. The experimental skyrmion numbers N_{sk} of featured supertopologies, marked by big dashed lines in (a2-h2), are shown in (a3-h3), respectively. The experimental N_{sk} values of selected protected baby skyrmions ("α","β","γ",) marked by black dashed lines are shown in (a3-d3), correspondingly.

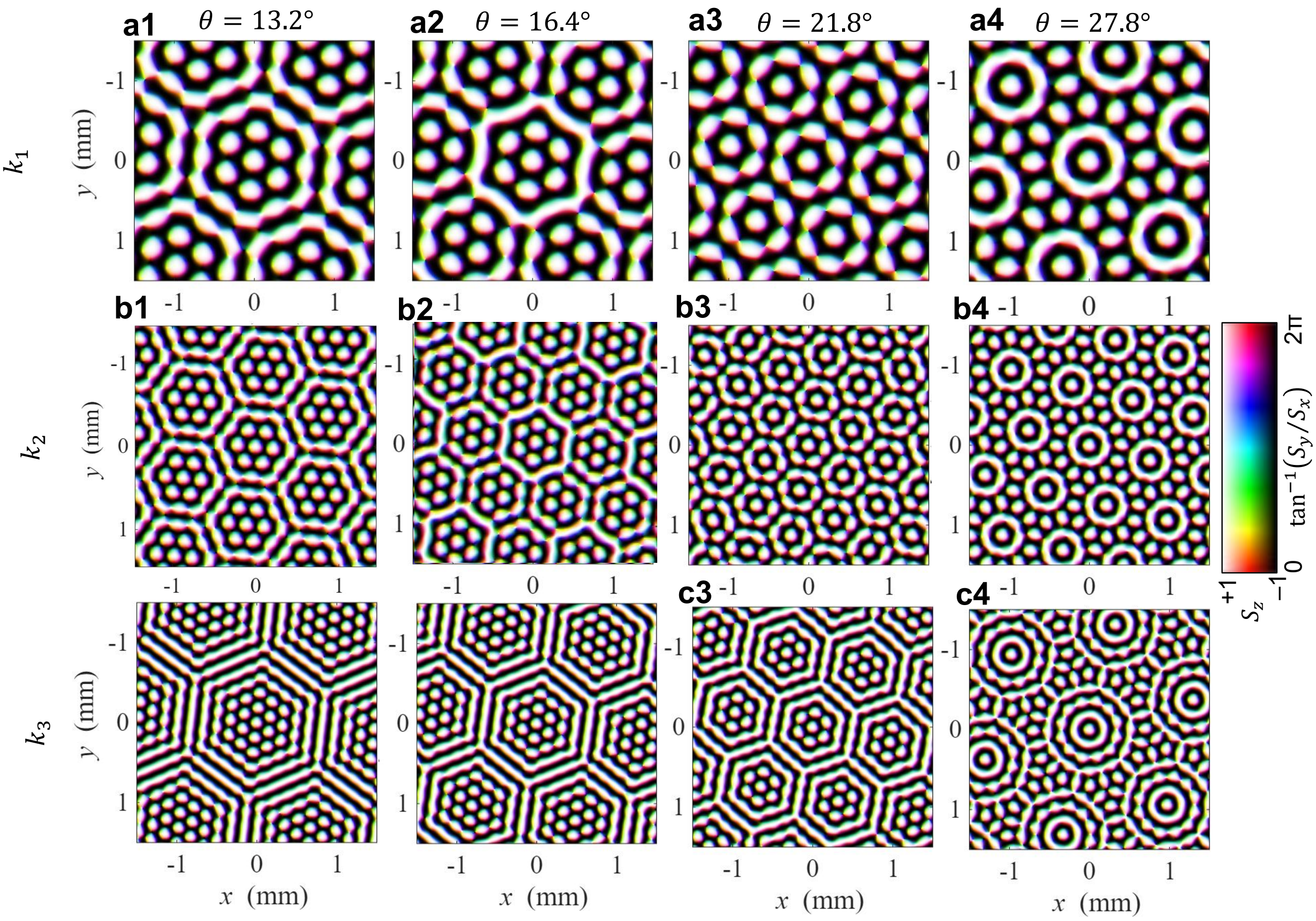

FIG. E5: **Hierarchical supertopology decomposition.** Decomposed skyrmion supertopologies generated from wavevectors (a1-a4)k_1, (b1-b4) k_2 and (c1-c4) k_3 obtained from the Fourier transform of Stokes vectors of C_6 skyrmionium lattices twisted at $\theta = 13.2°,\ 16.4°,\ 21.8°$, and $27.8°$, respectively.

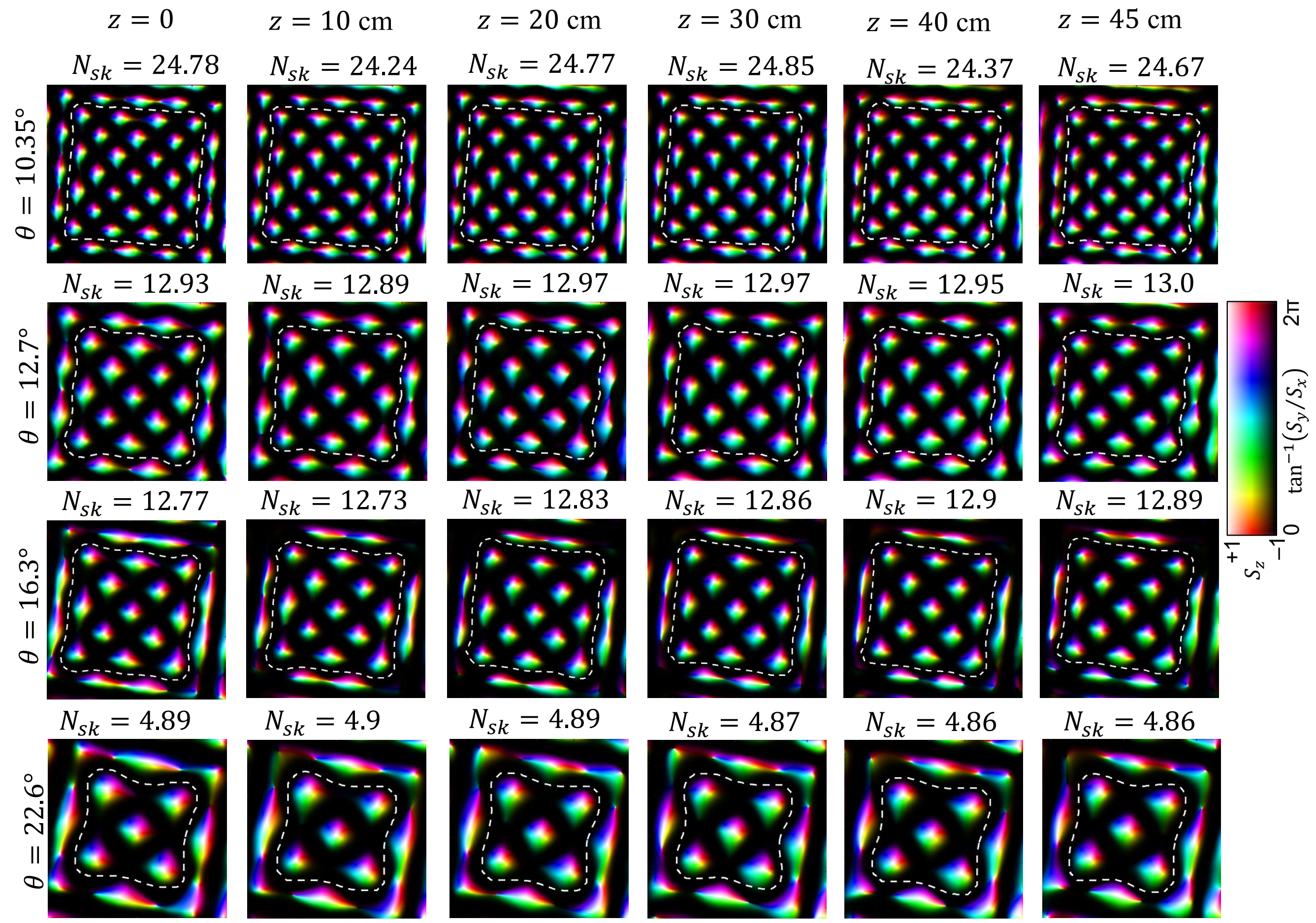

FIG. E6: **Experimental results for stable propagation of C_4 skyrmion-cluster superlattices.** Stable propagation of skyrmion-cluster superlattices obtained at twist angles $\theta = 10.35°$ (first row), $\theta = 12.7°$ (second row), $\theta = 16.3°$ (third row), and $\theta = 22.6°$ (fourth row).

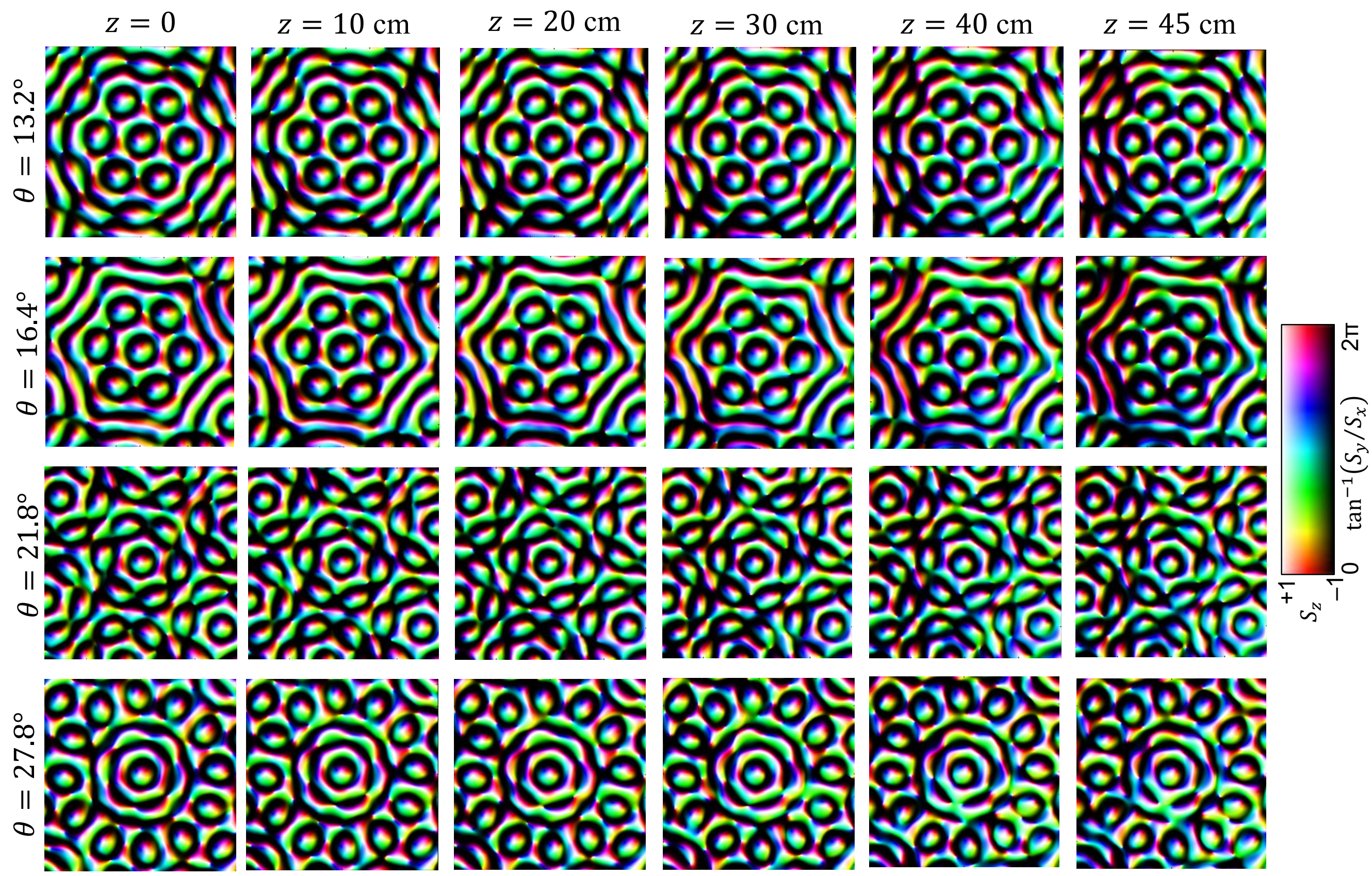

FIG. E7: **Experimental results for stable propagation of C_6 super topologies.** Stable propagation of skyrmionium supertopologies obtained by twisting two skyrmionium lattices at twist angles $\theta = 13.2°$ (first row), $\theta = 16.4°$ (second row), $\theta = 21.8°$ (third row), and $\theta = 27.8°$ (fourth row).

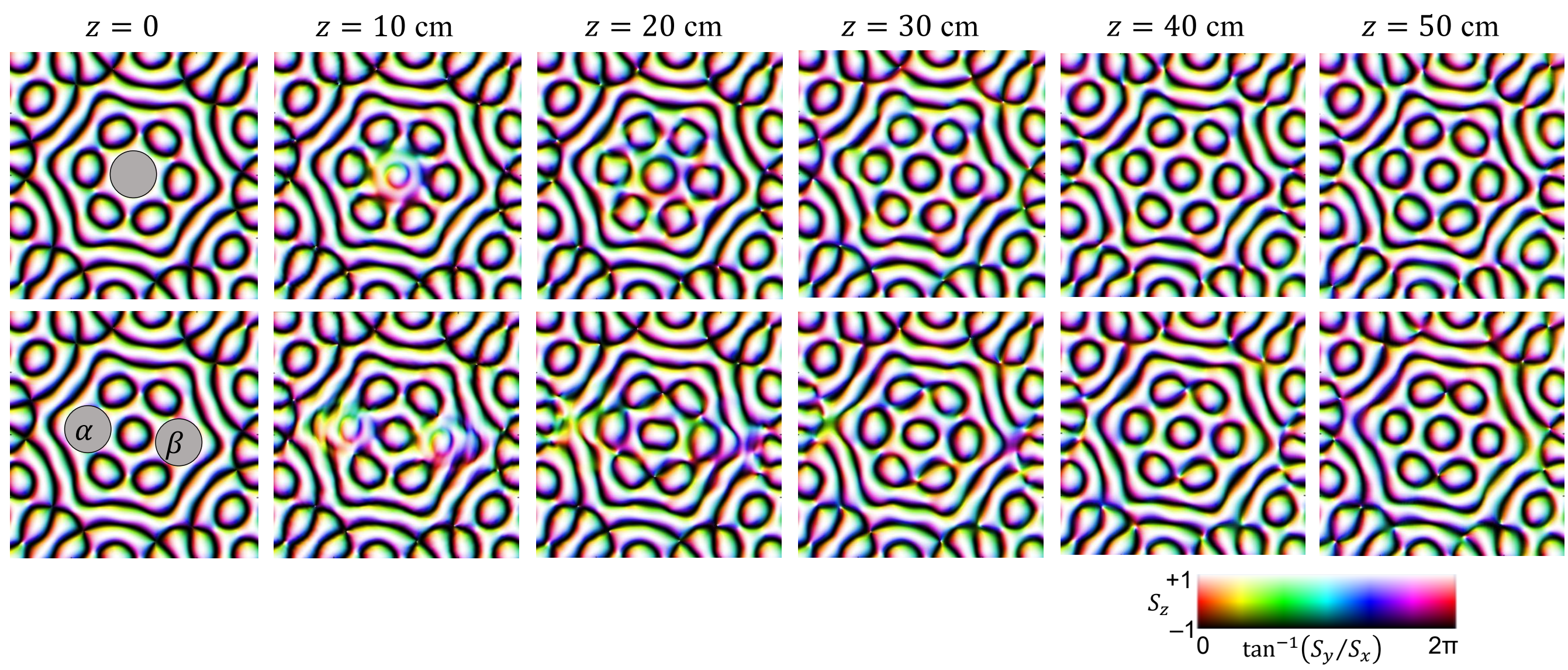

FIG. E8: **Simulation results for self-healing.** Self-healing of skyrmionium bag when central skyrmionium (first row) and two distinct skyrmioniums marked as α and β (second row) are blocked at $z = 0$.
