# Supplementary material for: Freespace “twistronics”: moiré structured light for optical supertopologies
Source: Light Sci Appl. 2026 Nov 6;15:396. doi: 10.1038/s41377-026-02533-w (PMC13740294; doi:10.1038/s41377-026-02533-w)
Supplement: Supplementary file 2 [file 41377_2026_2533_MOESM1_ESM.docx]

**Supplementary Materials for**

**Freespace “twistronics”: moiré structured light for optical supertopologies**

Vasu Dev^1, 2^ and Yijie Shen^1, 3, ∗^

*^1^ Centre for Disruptive Photonic Technologies, School of Physical and Mathematical Sciences, Nanyang Technological University, Singapore 637371, Republic of Singapore*

*^2^ Department of Physics, School of Advanced Sciences (SAS), Vellore Institute of Technology (VIT), Vellore 632014, Tamil Nadu, India.*

*^3^ School of Electrical and Electronic Engineering, Nanyang Technological University, Singapore 639798, Singapore*

*∗ yijie.shen@ntu.edu.sg*

**S1. Supertopological quasicrystal:**

**Fig. S1:** Generation of supertopological quasicrystal. (a,b,c) Out of plane Stokes vector $S_{z}$, topological texture, and skyrmion number density for the supertopological quasicrystal obtained from two $C_{6}$ skyrmionium lattices with inter layer twist $\theta={30}^{\circ}$. (d,e,f) Out of plane Stokes vector $S_{z}$, topological texture, and skyrmion number density for the supertopological quasicrystal obtained from two $C_{4}$ skyrmion lattices with inter layer twist $\theta={45}^{\circ}$.

**S2. Off-center twisting of topological lattices:**

**Fig. S2:** **Off-center twisting of topological lattices.** (a) Selected off-center high-symmetry points and in a skyrmionium lattice$C_{1}=$ **a**/2 and $C_{2}=$ (2**a** - **b**)/3, where **a** and **b** denote the primitive lattice vectors. (b,c) Skyrmionium-bag superlattice containing 10 baby skyrmioniums and (d,e) skyrmionium-cluster superlattice containing three baby skyrmioniums, obtained by twisting the constituent lattices about $C_{1}$ at $\theta={13.2}^{\circ}$ and $\theta={21.8}^{\circ}$ respectively. (f,g) Skyrmionium-bag superlattice containing 12 baby skyrmioniums and (h,i) three-baby-skyrmionium supertopological structure obtained by twisting the lattices about $C_{2}$ at $\theta={13.2}^{\circ}$ and $\theta={21.8}^{\circ}$ respectively.

To investigate the effect of off-center twisting, we consider two high-symmetry points of the skyrmionium lattice, $C_{1}=$ **a**/2 and $C_{2}=$ (2**a** - **b**)/3, where **a** and **b** are the primitive lattice vectors, as shown in Fig. S2 (a). To investigate the effect of off-center twisting, the second lattice is twisted by an angle $\theta$ about $C_{1}$ or $C_{2}$.

When the lattices are twisted about $C_{1}$, a skyrmionium-bag superlattice containing 10 baby skyrmioniums is obtained at $\theta={13.2}^{\circ}$, as shown in Figs. S2 (b, c). At $\theta={21.8}^{\circ}$, a skyrmionium-cluster superlattice containing three baby skyrmioniums (Figs. S2 (d, e)) is obtained. Similarly, twisting about $C_{2}$ produces a skyrmionium-bag superlattice containing 12 baby skyrmioniums at $\theta={13.2}^{\circ}$ (Figs. S2 (f,g)), whereas a three-baby-skyrmionium configuration is obtained at $\theta={21.8}^{\circ}$ (Figs. S2 (h,i)).

These results demonstrate that, even at the same commensurate twist angle, changing the rotation center modifies the relative interference of the constituent lattices and can therefore alter the number and spatial arrangement of the topological elements within the supercell. Off-center twisting thus provides an additional translational degree of freedom, complementary to the twist angle, for controlling the generated optical supertopologies.

**S3. Experimental setup:**

We describe here the experimental setup used to generate the optical supertopologies. To demonstrate the proof of concept, we generate the supertopologies through complex-amplitude modulation of two orthogonally polarised monochromatic beams using computer-generated holograms displayed on the two halves of the spatial light modulator (SLM). A HOLOEYE ERIS SLM with resolution $1980\times1200$ and pixel pitch $8 \mu m$ is used. The finite transverse aperture of the generated fields imposes a practical limit on the minimum accessible twist angle. Since the moiré supercell size increases rapidly as the relative twist angle decreases. Therefore, in the present work we restrict the investigated commensurate configurations to $\theta>{10}^{\circ}$, for which complete supercells can be adequately resolved and reconstructed. This value represents a practical criterion for the present experimental configuration rather than a fundamental lower limit of freespace supertopologies. Smaller twist angles may be accessed by increasing the usable aperture or rescaling the constituent lattice. The diagonally polarised laser beam (wavelength $\lambda$= 532 nm) is split into horizontal and vertical components by a polarising beam splitter (PBS1) and two mirrors (M1 and M2) arranged in a Sagnac interferometric configuration. To increase the modulation efficiency, we directly encode holograms for twisted lattice fields, rather than generating them through arrays of $2N$Gaussian beamlets ($N=3, 4,$ or $6$). As the SLM modulates only horizontally polarised light beam, a small half-wave plate (HWP) is placed immediately before the second half of the SLM to rotate the polarisation appropriately, as shown in Fig S3(a) and S4.

After reflection from the SLM, the modulated beams are recombined using a second Sagnac interferometer formed by PBS2 and mirrors M4 and M5. The resulting beam is then converted into the right- and left-circular polarisation basis using a quarter-wave plate (QWP). For the reconstruction of the full polarisation field, the Stokes parameters are obtained from six intensity measurements corresponding to six analyser settings, as shown in Fig. S3(b).

**FIG. S3:** (a) Experimental setup for the generation of supertopologies. (b) Measured intensities corresponding to six different polarisations for stokes vector reconstruction. PBS1, PBS2 polarisation beam splitters; $\lambda/2$, HWP, and QWP, half wave plate quarter wave plate for $\lambda=532$ nm; M1-M5, mirrors; SLM, spatial light modulator.

**FIG. S4:** Picture of experimental setup to generate supertopologies obtained from freespace twistronics.

**S4. Effect of correction term** $\boldsymbol{\delta}$**:**

**Fig. S5:** Effect of correction term $\delta$ on the skyrmion number calculation in the C4 skyrmion cluster superlattice obtained at $\theta={12.66}^{\circ}$.

Here we investigate the role of a small correction term $\delta$ in calculating the skyrmion number of skyrmion-cluster superlattices. As the Gaussian (RCP) lattice and the vortex (LCP) lattice in C_4_ skyrmion lattice do not map one-to-one, the superposition produces isolated transverse points with intensity nulls (electromagnetic “dark spots”). These intensity nulls affect the out-of-plane Stokes component $S_{z}$, leading to an incorrect evaluation of the skyrmion number. To remove this artefact, we add a small constant $\delta$ to $S_{z}$ and renormalize it with magnitude of total Stokes vector. Figure S5 shows that introducing $\delta$ does not modify the topological features of the generated supertopologies. For very small $\delta$, residual intensity nulls lead to an inaccurate skyrmion number, whereas increasing $\delta$ causes the total skyrmion number to rise and then saturate at the correct value of 13. We find that $\delta=3\%$ of the total intensity provides more than 98% accuracy for all investigated twist angles $\theta={10.35}^{\circ}$, ${12.66}^{\circ}$, ${16.26}^{\circ}$, and ${22.61}^{\circ}$. We therefore use as a common correction term for all C_4_-based supertopologies considered in this work.

The correction acts as a weak spatially uniform background that removes the isolated intensity-null points where the normalized Stokes vector is not well-defined. Introducing ($\delta$) does not modify the LCP and RCP polarization basis vectors and therefore orthogonality between LCP and RCP is preserved. Since the correction is spatially uniform, it also does not change the spatial wavevectors, relative twist angle, or the underlying symmetry or periodicity of the moiré superlattice. It only produces a small local modification of the Stokes-vector distribution around the electromagnetic dark spots.

**S5. Twisting in C_3_ meron lattices:**

Here we present the results for C_3_ meron topological lattices. For N = 3, the underlying topological texture forms a C_3_ meron lattice with a triangular unit cell. When two such lattices are twisted, meron-cluster superlattices emerge at commensurate twist angles. Figures S6(a–d) show the topological textures obtained for twist angles $\theta=13.2^{o}$*,* $16.4^{o}$*,* $21.8^{o}$*,*and $27.8^{o}$*.* As evident, the size of the superunit cell decreases as the twist angle increases. The corresponding skyrmion-number density distributions, shown in Figs. S6 (f–i), clearly show the meron cluster superlattice structures. For a twist angle of $\theta={60}^{o}$, a distinct configuration appears that closely resembles a skyrmionium lattice, as shown in Figs. S6(e) and S6(j).

**Fig. S6: C3 meron lattices.** (a-e) Topological texture, and (f-j) skyrmion number density obtained when two C_3_ meron lattices are twisted with angle at $\theta=13.2^{\circ}$, $16.4^{\circ}$, $21.8^{\circ}$, $27.8^{\circ}$, and $60^{\circ}$.

**S6. Effect of topological charge:**

**Fig. S7**: Effect of topological charge. (a) Intensity and (b) phase distributions of scalar C_6_ lattice for topological charge 2. (c) Topological lattice and (d) superlattice obtained at twist angle $\theta={16.4}^{\circ}.$

**S7. Hierarchical supertopology decomposition for C_4_:**

Here we present the Fourier-space decomposition of a twisted C_4_ skyrmion superlattice. As an example, we consider the configuration when two C_4_ skyrmion lattices are twisted by an angle of $\theta={16.26}^{o}$. Owing to the periodicity of the Stokes vector, its Fourier transform consists of discrete spatial-frequency components. Figure S8(a) shows the Fourier transform of the in-plane Stokes vector, where the Fourier components are organised into four concentric rings with radial spatial frequencies $k_{1}$, $k_{2}$, $k_{3}$, and $k_{4}$. Each ring corresponds to a sublattice that contributes to the overall twisted skyrmion superlattice.

The sublattices associated with $k_{1}$ and $k_{3}$ each containing four Fourier components, reconstruct C_4_ meron lattices with different lattice constants, as shown in Figs. S8(b) and S8(e). The ring at wavevector $k_{2}$ contains eight Fourier components, corresponds to a texture equivalent to two C_4_ meron lattices twisted by $\theta={16.26}^{o}$, as shown in Fig. S8(c). The outer ring at wavevector $k_{4}$consists of twelve Fourier components and represents a twisted trilayer texture in real space, as illustrated in Fig. S8(d).

**Fig. S8:** Simulation results for hierarchical decomposition for twisted C4 lattices. (a) Fourier transform of in plane stokes vector of twisted C4 skyrmion lattices at $\theta={16.26}^{o}$, where Fourier components are arranged in four concentric rings of radial spatial frequencies $k_{1}-k_{4}$. (b-e) Hierarchical real-space textures and skyrmion number density of reconstructed meron lattices and meron cluster in C4 geometry for frequencies $k_{1}-k_{4}$.

**S8. Hierarchical supertopology decomposition for C_3_:**

Here we present the Fourier-space decomposition of twisted meron-cluster superlattices. As an example, we analyse the in-plane Stokes vector of a C_3_ meron lattice twisted by $\theta={21.8}^{o}$. For an untwisted C_3_ meron lattice, the Fourier transform of the Stokes vector contains six discrete frequencies with the same radial wavenumber and therefore cannot be decomposed further into distinct sublattices. In contrast, for the twisted case we find that the Fourier spectrum consists of four distinct rings of spatial-frequency vectors, labelled $k_{1}-k_{4}$, as shown in Fig. S9(a). Selective inverse Fourier transforms of these rings yield four meron sublattices.

The frequency rings $k_{1}$, $k_{2}$, and $k_{4}$ each contain six equally spaced Fourier components, and the corresponding sublattices reconstruct meron lattices with different lattice constants, as shown in Figs. S9(b–d). The ring at $k_{3}$ contains twelve Fourier components arranged in two sets of six, with one set rotated by $\theta={21.8}^{o}$ relative to the other. The resulting sublattice, shown in Fig. S9(e), exhibits a unique twisted topological texture that closely resembles a skyrmion-bag configuration.

**Fig. S9:** Simulation results for hierarchical decomposition for twisted C_3_ lattices. (a) Fourier transform of in plane stokes vector of twisted C_3_ meron lattices at $\theta={21.8}^{o}$, where Fourier components are arranged in four concentric rings of radial spatial frequencies $k_{1}-k_{4}$. (b-e) Hierarchical real-space textures and skyrmion number density of corresponding reconstructed meron lattices and meron cluster in C_3_ geometry for frequencies $k_{1}-k_{4}$.

**S9. Effect of amplitude:**

**Fig. S10:** **Effect of relative amplitude on the supertopological texture and its hierarchical Fourier decomposition.** Real-space Stokes-vector texture and reconstructed components corresponding to the spatial-frequency groups $k_{1}$, $k_{2}$, and $k_{3}$ for representative amplitude ratios $\mu<1$, $\mu=1$, and $\mu<1$ (top to bottom). Variation of $\mu$modifies the local Stokes-vector distribution while preserving the characteristic supertopological structure. The effect of amplitude imbalance is most pronounced in the component $k_{1}$, whereas the $k_{2}$, and $k_{3}$ components exhibit comparatively smaller changes.

The parameter $\mu=A_{2}/A_{1}$ denotes the relative amplitude of the two orthogonally polarized fields forming the topological lattice. To investigate the effect of $\mu$, we calculate the complete supertopological texture and its hierarchical Fourier-space components for representative cases of $\mu<1$*,* $\mu=1$*,* and $\mu<1$, as shown in Fig S10.

As $\mu$ is varied, the relative weighting of the Stokes-vector components changes, leading to a modification of the local polarization texture. Despite these local changes, the characteristic real-space supertopological structure remains distinguishable over the investigated range of $\mu$.

The Fourier-space decomposition further reveals that amplitude imbalance does not change the characteristic spatial-frequency positions $k_{1}$*,* $k_{2}$*,* and $k_{3}$. However, change in $\mu$ modifies the relative contribution of these Fourier components to the reconstructed Stokes texture. In particular, the $k_{1}$ component exhibits a more pronounced dependence on $\mu$, whereas topological textures for $k_{2}$*,* and $k_{3}$ remain comparatively less affected. Therefore, different hierarchical components of the supertopology exhibit different sensitivities to relative-amplitude imbalance.

Overall, these results demonstrate that the relative amplitude provides an additional degree of freedom for tuning the local Stokes-vector distribution and the hierarchical composition of the supertopological field, while the overall supertopological structure remains robust against moderate amplitude variations.

**S10. Non-coaxial twisting:**

**Fig. S11:** Non-coaxial twisting of topological lattices. (a) Fourier plane and (b) lattice plane of non-coaxial lattices.

For non-coaxial twisting, we introduce a transverse displacement of the spatial-frequency distribution of one lattice with respect to the other in the Fourier plane, as shown in Fig. S11 (a). This displacement introduces a transverse wave-vector offset ($\Delta x, \Delta y)$ and consequently tilts the propagation axis of one lattice with respect to the other, as illustrated in Fig. S11 (b). Now, each constituent topological lattice can still retain its nondiffracting characteristics along its own propagation axis. However, when the total field is considered with respect to a single laboratory propagation axis, the longitudinal projections of their wavevectors are generally different, resulting in the propagation-dependent variation in the relative phase. Hence, propagation invariance of the supertopology about the single laboratory axis is generally no longer expected.

**Fig. S12:** Simulation results for non-coaxial twisting. Propagation of the skyrmionium bag topology $(\theta={16.4}^{\circ})$for transverse Fourier-plane displacement of $\Delta x=\omega_{0}$ (first row) and $\Delta x=2\omega_{0}$ (second row). The larger Fourier-plane displacement in the second case corresponds to a larger relative tilt between the propagation wave vector of the two topological lattices.

To investigate the effect of non-coaxial twisting, we consider two cases of transverse displacement of the spatial-frequency distribution in the Fourier plane, $\Delta x=\omega_{0}$ and $\Delta x={2\omega}_{0}$, as shown in the first and second rows of Fig. S12. The larger Fourier-plane displacement produces a larger relative tilt between the propagation axes of the two lattices. Each lattice preserves its nondiffracting characteristics along its own propagation axis. However, the relative tilt results in different longitudinal wave-vector components with respect to the common laboratory z-axis. Consequently, the two lattices accumulate different propagation phases during the propagation. Owing to the change in relative phase, the interference condition along z changes. Thus, the initially formed skyrmionium bag deforms during propagation. As the relative phase changes with z, the deviation from the initial supertopological structure becomes more pronounced over the propagation range shown in Fig. S12.
